# Ciprofloxacin Poly(β-amino ester) Conjugates Enhance Antibiofilm Activity and Slow the Development of Resistance

**DOI:** 10.1021/acsami.3c14357

**Published:** 2024-01-30

**Authors:** Karolina Kasza, Brogan Richards, Sal Jones, Manuel Romero, Shaun N. Robertson, Kim R. Hardie, Pratik Gurnani, Miguel Cámara, Cameron Alexander

**Affiliations:** † Division of Molecular Therapeutics and Formulation, School of Pharmacy, University of Nottingham, Nottingham NG7 2RD, U.K.; ‡ National Biofilms Innovation Centre, School of Life Sciences, Biodiscovery Institute, University Park, University of Nottingham, Nottingham NG7 2RD, U.K.; § Department of Microbiology and Parasitology, Faculty of Biology-CIBUS, Universidade de Santiago de Compostela, Santiago de Compostela 15782, Spain; ∥ UCL School of Pharmacy, University College London, 29-39 Brunswick Square, London WC1N 1AX, U.K.

**Keywords:** polymer antimicrobials, antibiotic resistance, biofilms, quorum sensing, polymer–drug conjugates, combination anti-infectives

## Abstract

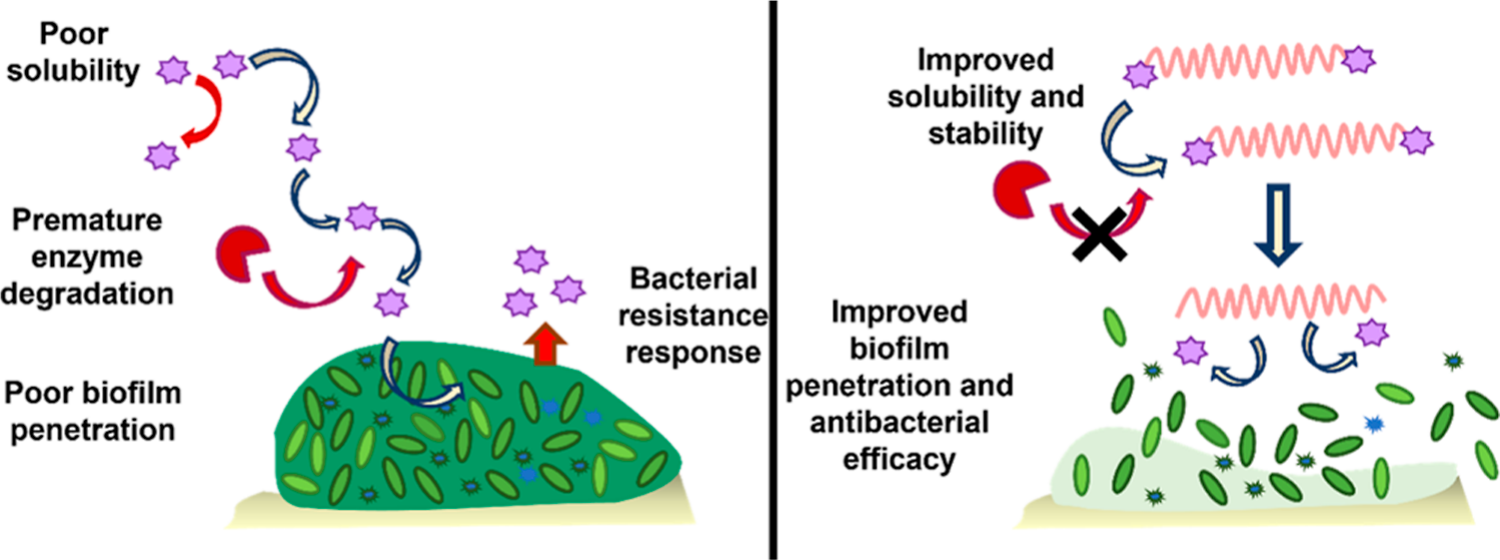

To tackle the emerging antibiotic resistance crisis, novel antimicrobial approaches are urgently needed. Bacterial biofilms are a particular concern in this context as they are responsible for over 80% of bacterial infections and are inherently more recalcitrant toward antimicrobial treatments. The high tolerance of biofilms to conventional antibiotics has been attributed to several factors, including reduced drug diffusion through the dense exopolymeric matrix and the upregulation of antimicrobial resistance machinery with successful biofilm eradication requiring prolonged high doses of multidrug treatments. A promising approach to tackle bacterial infections involves the use of polymer drug conjugates, shown to improve upon free drug toxicity and bioavailability, enhance drug penetration through the thick biofilm matrix, and evade common resistance mechanisms. In the following study, we conjugated the antibiotic ciprofloxacin (CIP) to a small library of biodegradable and biocompatible poly(β-amino ester) (PBAE) polymers with varying central amine functionality. The suitability of the polymers as antibiotic conjugates was then verified in a series of assays including testing of efficacy and resistance response in planktonic Gram-positive and Gram-negative bacteria and the reduction of viability in mono- and multispecies biofilm models. The most active polymer within the prepared PBAE-CIP library was shown to achieve an over 2-fold increase in the reduction of biofilm viability in a *Pseudomonas aeruginosa* monospecies biofilm and superior elimination of all the species present within the multispecies biofilm model. Hence, we demonstrate that CIP conjugation to PBAEs can be employed to achieve improved antibiotic efficacy against clinically relevant biofilm models.

## Introduction

The increasing prevalence of antibiotic-resistant pathogens poses a growing challenge in community healthcare due to excessive antimicrobial consumption in both humans and livestock.^1^ Bacterial biofilms are of particular concern as they are responsible for up to 80% of human infections and can contribute to the development of chronic infections, characterized by persistent inflammation and tissue damage.^2,3^ The efficacy of conventional antibiotic therapies to fight bacterial infections is hindered by issues such as limited drug solubility, systemic toxicity, premature degradation, and susceptibility to resistance development.^4^ Moreover, bacterial biofilms have been shown to exhibit high levels of resilience to antimicrobial treatments attributed to several factors, including reduced drug diffusion through the dense exopolymeric biofilm matrix and upregulation of antimicrobial resistance machinery through phenotypic changes in gene expression.^5−7^ Furthermore, the polymicrobial nature of most biofilms means different species, including fungi, bacteria, and viruses, can synergistically interact and coexist leading to recalcitrant infections.^8^ It is therefore concerning, considering the rise in antibiotic resistance and the limitations of current therapies, that fewer than one new antibiotic reaches the worldwide market each year on average.^9,10^ This is partially due to the substantial costs linked with new drug development and approval, combined with low financial reward associated with antimicrobial sales.^2,11^ Existing antibiotic formulation and derivatization offer an alternative approach to this problem by reducing the time and cost associated with new therapy development, while offering several improvements upon the current therapies by enhancing drug efficacy and reducing adverse side effects.^12^ This is particularly the case for bacterial biofilms, where drug delivery can improve upon antibiotic diffusion through the exopolymeric matrix and avoid its premature deactivation by preventing drug binding to matrix components and its enzymatic deactivation.^13−15^ To date, a plethora of materials have been reported for drug delivery, including lipids, inorganic substances, and metals; however, the use of polymeric systems offers the highest versatility, enabling their fine-tuning to achieve optimal drug activity.^2,16,17^ Polymer systems can deliver their selected drug cargo either through drug encapsulation or conjugation within a polymeric micelle or by therapeutic conjugation to a fully soluble polymer chain composed of a single backbone. A substantial advantage associated with the latter is their flexible random coil structure, which allows them to easily penetrate gaps smaller than their hydrodynamic diameter.^18^ Moreover, polymer conjugation has been shown to evade drug adsorption to the biofilm matrix and bacterial cell surfaces, reported as negatively charged at physiological pH and transitioning to a positive charge once in the decreasing pH of the biofilm.^19^ We therefore hypothesized that the use of polymer–antimicrobial conjugates could show superior biofilm penetration in addition to further advantages including an improvement of drug solubility and bioavailability, reduction of toxicity, evasion of common resistance mechanisms, and controlled antimicrobial release at the target site.^20,21^

Antibiotic attachment to a polymer scaffold has to date been reported for several polymers including poly(ethylene glycol) (PEG),^22,23^ poly(2-oxazolines),^12,24,25^ poly(*N*-isopropylacrylamide),^26^ poly(methacrylates),^27^ and polypeptides^28^ with the antibiotic either permanently attached to the polymer backbone (antibiotic activity retained following attachment to polymer) or gradually released through cleavable linkers (antibiotic converted to a pro-drug through polymer attachment) with improvements in antibiotic efficacy reported in each case. Despite these promising results, the materials used to date have been either limited by their poor biodegradability or challenging to synthesize. Considering the significant advantages associated with the use of biodegradable materials including high tissue compatibility and minimized toxicity, the application of such systems warrants further exploration.^29^ Moreover, to enhance polymer–drug conjugate efficacy, the polymers need to be easily synthetically modified to explore optimal chemical functionality. An additional limitation associated with many of the reports to date involved a focus on planktonic cell efficacy, with the more clinically relevant activity in bacterial biofilms relatively unexplored. This is further surprising considering the promising efficacy reported by Du et al. following tobramycin conjugation to PEG, where a substantial improvement in antibiofilm activity was observed following antibiotic attachment to the polymer chain.^22^ Considering bacterial biofilms are responsible for up to 80% of human infections, the lack of frequent biofilm testing poses a question regarding the clinical applicability of linear polymer–drug conjugates and requires further investigation.^2,3^

We therefore prepared a novel library of drug–polymer conjugates, where the wide-spectrum antibiotic ciprofloxacin (CIP) was conjugated to a small chemical library of poly(β-amino ester) (PBAE) polymers, selected due to their inherent biocompatibility, biodegradability, responsiveness, and structural versatility.^30^ Synthesized through a one-pot aza-Michael addition of primary or secondary amines with diacrylates, PBAEs contain tertiary amine groups enabling their use for carrying negatively charged cargo or as pH-responsive materials, leading to their frequent application in gene delivery.^30−34^ Nonetheless, PBAE use for antimicrobial delivery has to date been limited, with applications focused on polymer micelle formation and drug encapsulation.^35−37^ Antimicrobial conjugation to the polymer using a cleavable linker was reported for triclosan in PEG–PBAE micelles;^38^ however, the administration of linear polymer–antibiotic conjugates in nonparticle form has, to our knowledge, not been reported to date and was therefore investigated.

**Figure 1:**
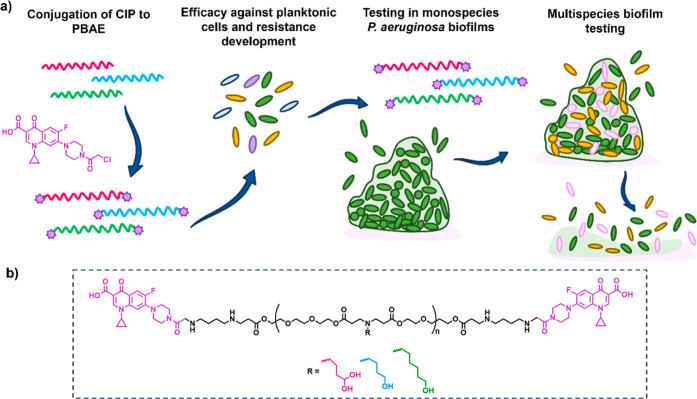
Experimental outline. (a) Schematic to show evaluation of PBAE–CIP conjugates (shown as red, blue, and green lines with CIP denoted by purple stars) in antimicrobial assays in planktonic conditions and in single species and multispecies biofilms; (b) structural outline of the PBAE scaffold used, with variation of the tertiary amine side chains denoted as R.

Hence, a CIP derivative was attached to a small chemical library of PBAEs based on hydrophilic monomer tetra(ethylene glycol) diacrylate (TEGDA) combined with three amines with varying hydrophobicity and alcohol group content (Figure 1). Following an initial assessment of planktonic activity and resistance development, the three prepared polymer scaffolds were then tested on biofilm models of *Pseudomonas aeruginosa* (monospecies), and this organism was grown with *Staphylococcus aureus* and *Candida albicans* (multispecies), in each case improving upon free CIP activity.

## Materials and Methods

### Materials

TEGDA, 3-amino-1,2-propanediol (3APD), 3-aminopropanol (3AP), 5-aminopropanol (5AP), 1,4-diaminobutane, ciprofloxacin hydrochloride (CIP) (98% pure), triethylamine (TEA), chloroacetyl chloride, ammonium sulfate, sodium chloride, dimethyl sulfoxide (DMSO)-*d*_6_ (99.5% D atom), and chloroform-*d* (99.8% D atom) were obtained from Sigma-Aldrich and used without further purification.

#### Microbial Strains

Bacterial strains *Escherichia coli* (Gram-negative, CFT073HT^39^), *Pseudomonas aeruginosa* (Gram-negative, PAO1-L^40^), *S. aureus* (Gram-positive, SH1000^41^), *Staphylococcus epidermidis* (Gram-positive, RP62A^42^) and fungal strain *Candida albicans* (SC5314^43^) were used for the following assays.

#### Bacterial Media Preparation

For the solid agar plugs, synthetic sputum media 2 (SCFM2) was prepared as reported in literature^44^ at twice the initial concentration and combined with 3% technical agar (Sigma-Aldrich).

Solvents and other reagents were acquired from commercial sources and used as-received, unless stated otherwise.

### Instrumentation and Analysis

#### NMR Spectroscopy

Proton nuclear magnetic resonance (^1^H NMR) spectra were recorded on a Bruker DPX-400 spectrometer using dimethyl sulfoxide (DMSO)-*d*_6_ (99.5% D atom) or chloroform-*d* (99.8% D atom).

#### Size Exclusion Chromatography

A Polymer Laboratories PL-50 instrument equipped with a differential refractive index (DRI) was used for size exclusion chromatography (SEC) analysis. The system was fitted with 2× PLgel Mixed D columns (300 mm × 7.5 mm) and a PLgel 5 μm guard column. The eluent used was DMF with 0.1% LiBr. Samples were run at 1 mL min^–1^ at 50 °C. Poly(methyl methacrylate) standards (Agilent EasyVials) were used for calibration between 955,500 and 550 g mol^–1^. Analyte samples were filtered through a membrane with a 0.22 μm pore size before injection. Experimental molar mass (*M*_n,SEC_) and dispersity (*D̵*) values of synthesized polymers were determined by conventional calibration using Cirrus GPC software.

### Methods

#### 7-(4-(2-Chloroacetyl)piperazin-1-yl)-1-cyclopropyl-6-fluoro-4-oxo-1,4-dihydroquino-3-carboxylic Acid Synthesis

7-(4-(2-Chloroacetyl)piperazin-1-yl)-1-cyclopropyl-6-fluoro-4-oxo-1,4-dihydroquino-3-carboxylic acid (CIP-Cl) was synthesized as previously reported in literature.^24,45^ Ciprofloxacin (1 g, 3.02 mmol, 1 equiv) was suspended in 20 mL of dichloromethane. Triethylamine (0.305 g, 3.02 mmol, 1 equiv) was added at room temperature, and the reaction mixture was stirred for 15 min. Chloroacetyl chloride (0.53 g, 4.68 mmol, 1.55 equiv) was slowly added to the solution at 0 °C. The reaction was heated to room temperature and stirred for 24 h. The product was precipitated in diethyl ether (250 mL) under suction filtration, and the solid residue was purified by column chromatography (SiO_2_; CH_2_Cl_2_:MeOH = 20:1) to get the product as slightly yellow solid (49%, 1.8 mg, 4.4 mmol).

#### PBAE Synthesis

PBAEs were synthesized as previously reported in literature.^46^ TEGDA (2 g, 6.6 mmol) was mixed with the selected amine at a 1.1:1 molar ratio of monomer to amine in DMSO at 500 mg mL^–1^, and the reaction was performed under stirring in the dark at 90 °C for 24 h. Following reaction completion, the mixture was diluted (167 mg mL^–1^) and end-capped using 1,4-diaminobutane (0.5 M) at 25 °C for 24 h. The resulting polymer was purified in tetrahydrofuran (THF) and diethyl ether (1:9), and the solvent was removed under reduced pressure to yield a yellow, viscous liquid. Amine capping efficacy was assessed using ^1^H NMR with no acrylate peaks present following the capping steps. The final polymers were characterized by SEC and ^1^H NMR.

#### PBAE-CIP Synthesis

Amine-capped PBAEs (1 g, 1 equiv), CIP-Cl (4 equiv), and sodium hydrogen carbonate (4 equiv) were solubilized in a 50:50 mixture of acetonitrile (ACN) and dimethylformamide (24 mg mL^–1^ CIP-Cl final concentration), and the mixture was stirred (450 rpm, 25 mm stirrer bar) for 48 h. Following reaction completion, the polymers were solubilized in methanol (5 mL) and centrifuged to separate unreacted CIP-Cl, following which the solute was collected and centrifuged two more times. The resulting polymer solution in methanol was then washed with diethyl ether and the solvent removed under reduced pressure yielding the PBAE-mCTAs as yellow, viscous liquids. The final polymers were analyzed by SEC and ^1^H NMR.

#### CIP Quantification by HPLC

To quantify the amount of CIP attached to the PBAE chain, the polymers were degraded in a 50:50 mixture of DMSO and trifluoroacetic acid (TFA) and left under stirring for 3 h. The solution was then diluted to 1:10 in DMSO and encapsulation levels assessed by high-performance liquid chromatography (HPLC) (Agilent technologies 1200 series, USA).

Quantification of CIP was achieved using a C18 (4.6 × 250 mm; 5 μm) analytical column (ZORBAX Eclipse Plus). The UV detector was operated at 277 nm. The mobile phase consisted of a mixture of 2% acetic acid aqueous solution and ACN (84:16, v/v). The flow rate was set at 1.0 mL min^–1^ and injection volume at 10 μL.^47^

#### Polymer Titration

The assessment of polymer p*K*_a_ was measured using a titration method. Briefly, a sample of the polymer (2 mg) was dissolved in a sodium chloride solution (30 mL, 0.1 M), and the pH was adjusted to 10 through the addition of sodium hydroxide (0.1 M). The polymer solution was titrated with hydrochloric acid (0.1 M), and the pH value of solution was measured until a pH of 3 was achieved, with the solution titration profile plotted.

#### Bacterial Susceptibility Testing (IC_50_)

Polymer–CIP conjugates or free CIP (equivalent to 16 μg mL^–1^ CIP content) were dissolved in autoclaved MiliQ-grade water (1.00 mL), and a dilution series in Lysogeny Broth (LB) was prepared with halved concentrations in every following sample finishing at a CIP concentration of 0.0156 μg mL^–1^, giving a tested CIP concentration range between 8 and 0.0156 μg mL^–1^.

Single *P. aeruginosa*, *E. coli*, *S. aureus,* and *S. epidermidis* colonies were used to inoculate 5 mL LB broth in sterile universal tubes. Overnight cultures were grown at 37 °C in a shaking (200 rpm) incubator. After overnight growth, each culture was diluted to an optical density at 600 nm (OD_600_) value of 0.02 using LB. 100 μL of the diluted culture was treated with 100 μL of polymer or free drug solution diluted in LB, in triplicates, in a Costar 96-well plate. Samples were incubated at 37 °C for 24 h, and OD_600_ measurements for each well were recorded at T0 and T24 h. The results from the T24 h time point were then used to calculate the percentage of bacterial inhibition (eq 1). Percentage of bacterial survival was calculated by subtracting the percentage of inhibition from 100. Each experiment was repeated in triplicate.
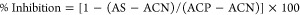
1Equation 1; Calculation of % inhibition, where *A*_CP_ is the absorbance of the positive control (no polymer), *A*_CN_ is the absorbance of the negative control (LB only), and *A*_S_ is the absorbance of the tested sample.^48^

IC_50_ values were calculated by plotting percentage survival against CIP concentration followed by calculating [inhibitor] vs normalized response with a variable slope in GraphPad Prism.

#### Resistance Development Studies in *P. aeruginosa* and *S. aureus*

Single *P. aeruginosa* and *S. aureus* colonies were used to inoculate 5 mL of LB broth in sterile universal tubes. Overnight cultures were grown at 37 °C in a shaking (200 rpm) incubator. After overnight growth, each culture was diluted to an OD_600_ value of 0.01 in LB (5 mL), and the selected polymer–CIP conjugate or free CIP treatment was added at its IC_50_ concentration, followed by overnight growth at 37 °C in a shaking (200 rpm) incubator. Following 24 h, the cultures were centrifuged (2 min, 10,000*g*) and the pellet resuspended in phosphate-buffered saline (PBS) and centrifuged again (2 min, 10,000*g*). The pellet was resuspended in LB and diluted to an OD_600_ value of 0.01 in LB (5 mL) followed by polymer/free CIP addition at its IC_50_ concentration and subsequent incubation at 37 °C in a shaking (200 rpm) incubator for 24 h. The procedure was repeated over a course of 8 days each time applying an IC_50_ concentration of either the polymer or free CIP, and on every occasion, 300 μL of the treated bacterial residue was retained as a 50:50 stock in glycerol (80%) and maintained at −80 °C.

#### Rolling Bioreactor Monospecies Biofilm Viability Studies

Mature 1 d old PAO1-L biofilms were used to characterize the effect of polymer and CIP treatments. Biofilms were grown as previously described in literature^49^ on round glass coverslips (13 mm diameter, #1.5 mm thickness) under dynamic conditions (20 rpm) in FAB medium^50^ consisting of glucose (10mM), ammonium sulfate (15.1 mM), sodium phosphate dibasic dihydrate (33.7 mM), potassium phosphate monobasic (22.0 mM), sodium chloride (51.3 mM), magnesium chloride (1 mM), calcium chloride (0.1 mM) and iron (III) chloride (3 μM); and inoculated with diluted (OD_600nm_ = 0.01) *P. aeruginosa* (PAO1-L) from overnight cultures in LB. Biofilms were cultivated at 30 °C for 24 h, following which the biofilms were washed in PBS to remove loosely attached cells and incubated for further 24 h in fresh medium supplemented with various treatments. These included free CIP at various concentrations (8–16 μg mL^–1^) and polymer–CIP conjugates at equivalent CIP concentrations. Biofilms exposed to each treatment were washed in PBS and the viability of attached cells evaluated by fluorescent staining using the LIVE/DEAD BacLight Bacterial Viability kit (Molecular Probes, Life Technologies) according to manufacturer instructions. Following staining, coverslips were rinsed with distilled water and imaged using an LSM700 AxioObserver (Carl Zeiss, Germany) confocal laser scanning microscope. Viable and non-viable biofilm biomass quantification from image stacks of biofilms was done with Fiji-ImageJ software. Live/dead ratios were established for each treatment and compared to those of untreated controls to obtain percentage changes in biofilm viability.

#### Static Mono- and Multispecies Biofilm Viability Studies

##### Biofilm Formation

Polycarbonate (PC) disks (isopore membrane filter [Sigma-Aldrich, Haverhill, UK]) with a pore size of 0.2 μm and diameter of 13 mm were sterilized for 15 min per side using short-wavelength ultraviolet light (UV-C) in a benchtop UV cabinet (Spectrolinker XL-1000 Series UV Cross-linker, town country). Agar (5 mL) consisting of 1× SCFM2 and 1.5% technical agar ([Oxoid, Cambridge, UK]) was added to wells of a 6 Well CELLSTAR Cell Culture Multiwell Plates (Greiner, Stonehouse, UK). Sterilised PC disks were added to the solid agar using forceps, and 10 μL of the desired microbial inoculum was then added to the center of the PC disks. For *P. aeruginosa* biofilms, a final inoculum of 1 × 10^4^ colony-forming units (cfu) mL^–1^ was added to the polycarbonate disk and incubated statically for 24 h at 37 °C before treatment.^100,101^ For polymicrobial biofilms, a final inoculum of 1 × 10^5^ cfu mL^–1^ for *C. albicans* and 1 × 10^6^ cfu mL^–1^ for *S. aureus* was initially inoculated onto the polycarbonate disk. Following a 24 h static incubation at 37 °C, the PC disks were moved to a fresh 1× SCFM2 with 1.5% technical agar plate, and 10 μL of a 1 × 10^4^ cfu mL^–1^*P. aeruginosa* inoculum was added and incubated for a further 24 h. The PC disks were then transferred to a fresh 1× SCFM2 with 1.5% technical agar plate containing 0.5 μg mL^–1^ with 10 μL of the corresponding concentration added directly to the top of the biofilms. The biofilms were incubated for further 24 h before disruption.

##### Microbial Enumeration

5 mm × 2.8 mm zirconium ceramic oxide beads (Fisherbrand, Loughborough, UK) were added to a 2 mL microcentrifuge tube with 1 mL of (3-(*N*-morpholino)propanesulfonic acid) (MOPS) minimal media without nitrogen and with succinate. A single biofilm was then added to the corresponding centrifuge tube and vortexed to remove the biofilm from the polycarbonate disk after which sterile forceps were used to remove the polycarbonate disks. The biofilms were then disrupted in a sonicating water bath (Fisherbrand, Loughborough, UK) for 25 min at a frequency of 37 kHz. The content of each tube was transferred to a corresponding 5 mL bijou containing 4 mL 1× MOPS minimal media. 10-fold dilutions were then performed from the sonicated samples using MOPS minimal media and 10 μL of each dilution and plated in triplicate on the desired selection agar. *P. aeruginosa* was plated on Pseudomonas isolation agar +4 μg mL^–1^ nystatin, *S. aureus* SH1000 on mannitol salt agar with 4 μg mL^–1^ nystatin, and *C. albicans* on sabouraud dextrose agar with 125 μg mL^–1^ tetracycline.

#### Assessment of Polymer Toxicity in Mammalian Cells

##### Human Cell Line Culture

A549s cells were grown in completed Dulbecco’s modified Eagle medium (DMEM, Sigma D6429) with 10% fetal bovine serum (FBS) (Sigma F7524) and 1% l-glutamine (G7513), kept at 37 °C and 5% CO_2_ and passaged twice weekly using trypsin (Sigma T3924).

##### Toxicity Assay

In a 96-well plate, 7000 (for 24 h assays) or 4000 (for 48 h assays) A549 cells were seeded per well in 200 μL of completed DMEM and incubated at 37 °C and 5% CO_2_ for 24 h to allow attachment to the bottoms of the wells. The medium was then replaced with treatments diluted 10-fold in completed DMEM to final concentrations of polymers equivalent to 4×, 2×, 1×, 0.5×, and 0.25× TEGDA-3APD-eCIP’s IC_50_ in planktonic *P. aeruginosa*, with 200 μL of treatment on each well, each condition in triplicate. Buffer (water), killed (1% final w/v Triton X 100), and completed DMEM conditions were included. Cells were incubated with these treatments at 37 °C and 5% CO_2_ for 24 h before PrestoBlue assay was performed–treated medium was replaced with 100 μL of PrestoBlue diluted 10-fold in PBS. The plate was incubated for 45 min before reading the fluorescence on TECAN Spark 10 M (excitation 535 nm, emission 615 nm). Results were normalized against killed control (0%) and medium control (100%) in Microsoft Excel and plotted as percentages in Graphpad Prism. Imaging of A549 cells following toxicity assays was conducted using an EVOS M5000 microscope.

## Results and Discussion

### Polymer Synthesis

**Figure 2:**
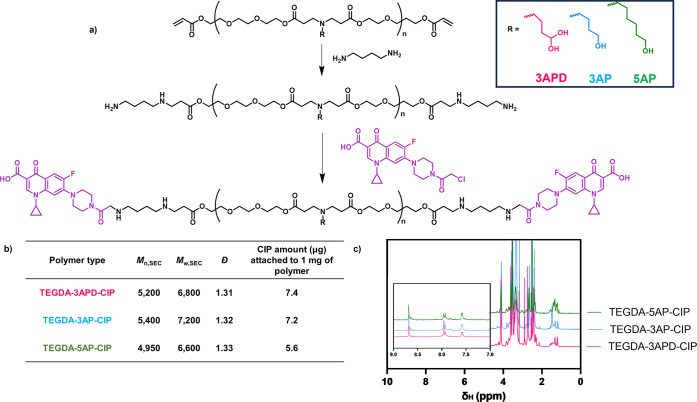
Synthesis and characterization of PBAE–CIP conjugates. (a) Synthetic scheme for synthesis of TEGDA-3APD-CIP, TEGDA-3AP-CIP, and TEGDA-5AP-CIP PBAE-CIP conjugates; (b) inset table with polymer characterization data including polymer number-average molecular weight (*M*_n_), weight-average molecular weight (*M*_w_), polydispersity (*D̵*), and quantification of CIP amount attached to each polymer chain; (c) ^1^H NMR spectra of three polymer–drug conjugates obtained, showing CIP peaks at around 8 ppm in each polymer.

The structure of PBAEs can be controlled by amine and diacrylate monomer selection for the step-growth polymerization that yields the polymer or end-group modifications of the final polymer chain. To ensure the solubility of the final PBAE–CIP conjugate, we selected a hydrophilic diacrylate monomer (TEGDA), which was used to produce PBAEs with three amines: 3-amino-1,2-propanediol (3APD), 3-aminopropanol (3AP), and 5-aminopropanol (5AP) to yield three different polymers: TEGDA-3APD, TEGDA-3AP, and TEGDA-5AP, respectively. Amine selection was based on increasing side chain hydrophobicity in order to promote insertion into the phospholipid bilayer in the bacterial membrane. Materials were synthesized utilizing a 1.1:1 diacrylate/amine ratio using previously reported conditions, with all polymers exhibiting residual acrylate signals in the ^1^H NMR spectra and a monomodal mass distribution with *M*_n,SEC_ = 7000 g mol^–1^.^46^ The terminal acrylates were then end-capped with an excess of 1,4-diaminobutane to yield amino functional PBAEs while avoiding any polymer–polymer coupling and enabling their subsequent functionalization with CIP. The final amino functional PBAEs displayed similar molar masses to their acrylate-terminated analogues while full amine functionalization was confirmed by the complete disappearance of the acrylate signals in the ^1^H NMR spectra (Figures S1 and S2). The amino-terminated PBAEs were then further functionalized with an amino-reactive CIP derivative (CIP-Cl) (Figure 2a).

### CIP–Polymer Conjugation

CIP contains two reactive sites for attachment to the polymer chain, either the carboxylic acid or the piperazine reactive handles at opposite ends of the drug molecule. Considering carboxylic acid functionality has been shown to be crucial for gyrase and topoisomerase IV binding.^51,52^ and therefore CIP activity, we decided to proceed with polymer attachment to the piperazine functional group with the aim of keeping the drug-binding site intact. A CIP amino-reactive derivative was synthesized using previously reported conditions by reacting the drug molecule with chloroacetyl chloride to yield CIP-Cl.^24^ Polymer conjugation was then performed with 4 equiv of CIP-Cl used per 1 equiv of selected polymer in the presence of sodium hydrogen carbonate as a base. Following purification in diethyl ether and methanol, the removal of unreacted CIP-Cl was verified by ^1^H NMR (Figure S1). A decrease in *M*_n,SEC_ to 5000 was observed following CIP conjugation, most likely as a result of poor solubility of higher-molar-mass chains in methanol when removing unreacted CIP-Cl (Figure S2). Successful CIP attachment was verified by ^1^H NMR through the presence of aromatic CIP peaks around 8 ppm (Figure 2c). The amount of CIP attached was then quantified by HPLC with drug amounts conjugated to each polymer variant (Figure 2b). We observed that the yield of the CIP-Cl conjugation step was low, with less than 10 μg of CIP attached per 1 mg of each polymer. This was much below the theoretical yield of over 100 μg of CIP per 1 mg of PBAE, with the poor yield resulting from the limited solubility of CIP-Cl in the reaction solvents. Considering the relatively low IC_50_ of CIP, the limited amount of antibiotic conjugated to the PBAE was deemed acceptable due to the low amounts of drug requiring administration.^53^

### Polymer–Drug Conjugate Activity against Planktonic Cells

**Figure 3:**
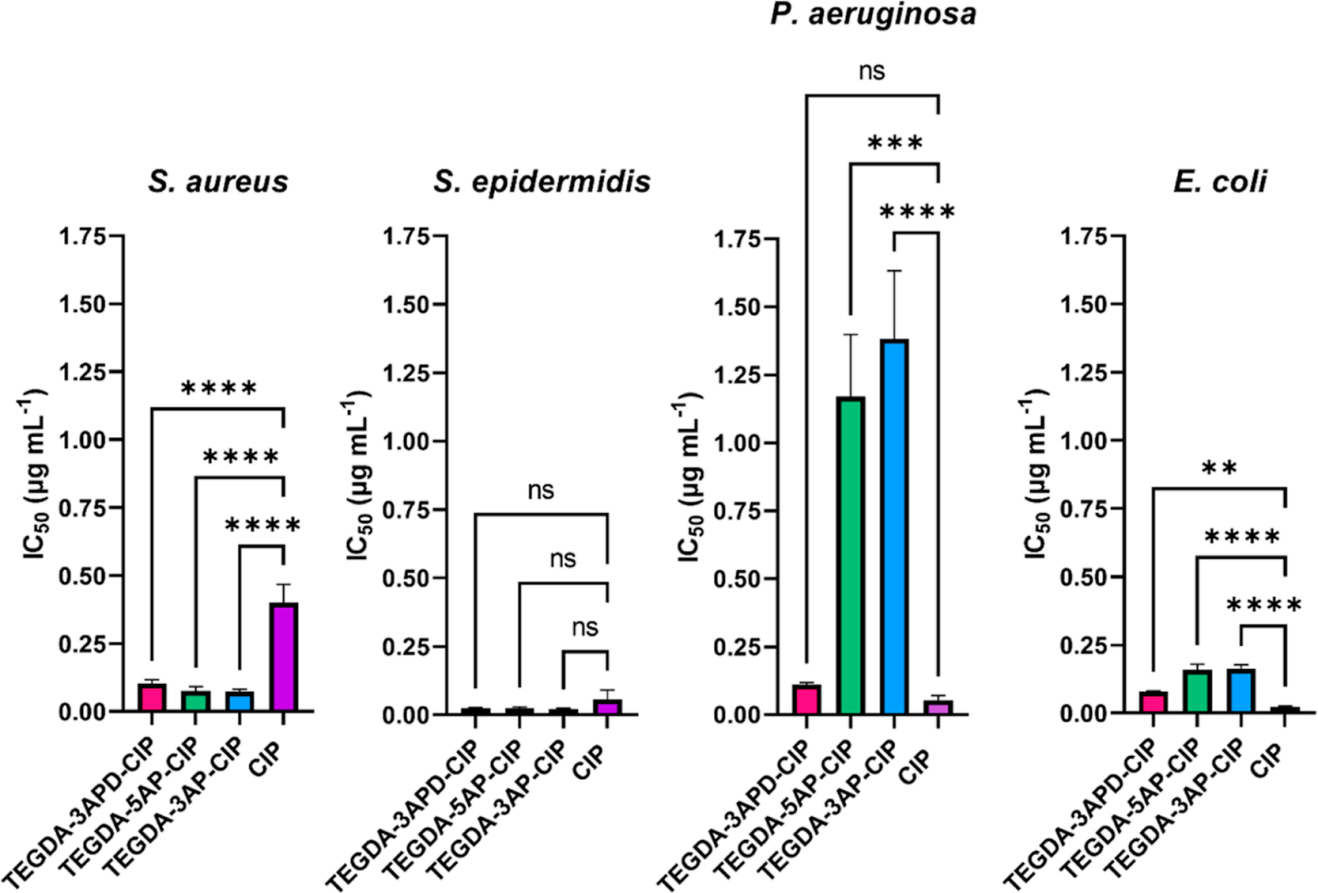
Efficacy against planktonic bacteria. IC_50_ (μg mL^–1^ of CIP attached) values for TEGDA-3APD-CIP (pink), TEGDA-5AP-CIP (green), and TEGDA-3AP-CIP (blue), against Gram-positive bacteria: *Staphylococcus aureus* (*S. aureus*) and *Staphylococcus epidermidis* (*S. epidermidis*) and Gram-negative bacteria: *Pseudomonas aeruginosa* (*P. aeruginosa*) and *E. coli* in comparison to the IC_50_ value (μg mL^–1^) of free CIP against each strain (purple). Calculated based on concentration curves in Figure S4. All measurements were performed in triplicate using biologically independent replicates, and the error bars represent the mean ± the standard deviation. Statistical testing was performed with a one-way analysis of variance (ANOVA) followed by a posthoc Tukey test to identify individual comparisons. Statistical significance is represented as *p < 0.05, **p < 0.01, ***p < 0.001, ****p < 0.0001.

The antimicrobial activity of the polymer–CIP conjugates was assessed against several clinically relevant Gram-negative and Gram-positive bacteria through the measurement of the half-maximum inhibitory concentration (IC_50_). These IC_50_ values were calculated based on the μg mL^–1^ of CIP attached to the polymer chain starting at concentrations corresponding to 8 μg mL^–1^ of CIP (equivalent to approximately 1.2 mg mL ^–1^ of the polymer mass). Following the attachment of CIP, the drug-conjugated polymers (TEGDA-3APD-CIP, TEGDA-3AP-CIP, TEGDA-5AP-CIP) showed high efficacy against the Gram-positive *S. aureus* with over a 3-fold decrease in IC_50_ observed for CIP attached to each polymer variant (IC_50_ values below 0.11 μg mL^–1^), compared to an equivalent concentration of the free drug control (IC_50_ of 0.40 μg mL^–1^) (Figure 3). For *S. epidermidis*, the other Gram-positive bacterium tested, the effect of polymer conjugation had a lesser impact on drug efficacy with comparable IC_50_ values of around 0.02 μg mL^–1^ observed for both the free drug and the PBAE-CIPs (Figure 3).

In Gram-negative bacteria, *Pseudomonas aeruginosa* and *E. coli*, more variation in activity was observed between the three polymers tested, with TEGDA-3APD-CIP outperforming TEGDA-3AP-CIP and TEGDA-5AP-CIP (Figure 3). This trend was particularly visible in *P. aeruginosa*, where TEGDA-3APD-CIP showed an IC_50_ of 0.11 μg mL^–1^, over 10-fold lower than the IC_50_ for TEGDA-3AP-CIP and TEGDA-5AP-CIP polymers, which were above 1 μg mL^–1^. The enhanced antimicrobial activity for the polymer with the diol functionality attached to the central amine within the polymer chain (i.e., TEGDA-3APD-CIP) went against our initial predictions in which we expected that the most hydrophobic amine side chain polymer (TEGDA-5AP-CIP) would show the highest antimicrobial activity. This was because the longer side chain might insert further into the bacterial cell walls and improve penetration. However, the lower efficacy observed for the TEGDA-3AP-CIP and TEGDA-5AP-CIP polymers in *P. aeruginosa* might be explained by its outer membrane permeability being significantly reduced (around 12- to 100-fold lower) compared to that of *E. coli*.^54,55^ It is possible therefore that the higher efficacy of TEGDA-3APD-CIP could result from this being a more hydrated polymer–drug conjugate and thus achieving a higher penetration through the outer membrane in *P. aeruginosa* via reduced interactions with cell wall components. In such a model, the mechanism of antibacterial action would therefore arise through delivering more of the attached antibiotic to the cytoplasm where it is active, rather than membrane disruption.

Moreover, the high activity of TEGDA-3APD-CIP could originate from different binding affinities of the three polymers to CIP target enzymes topoisomerase IV and gyrase.^56^ Depending on the type of bacteria, these two enzymes can either be the primary or secondary target for CIP, with DNA gyrase often the primary target in Gram-negative bacteria and the secondary target in Gram-positives.^57^ Previous reports by Schmidt et al. demonstrated that CIP conjugation to poly(2-oxazolines) lowered the antibiotic’s binding affinity to topoisomerase IV. The group further observed the induction of different structural modifications in *S. aureus* topoisomerase IV, with differences depending on the structure of the linker used to attach CIP to the polymer chain.^58^ Considering these findings, further analysis of the interactions of the PBAE-CIPs with the target proteins is required to elucidate the precise reasons behind the superior TEGDA-3APD-CIP efficacy.

For both *P. aeruginosa* and *E. coli,* the polymer–CIP conjugates showed a lower efficacy than that of the free drug control, with the IC_50_ of TEGDA-3APD-CIP being, respectively, 2-fold and 3-fold higher. The substantial difference in the activity of polymer–CIP conjugates against Gram-positive and Gram-negative bacteria can be explained by the presence of an additional cell membrane in the latter. Considering that the PBAE–CIP conjugates and free drug need to access the cytoplasm to be active, the presence of an additional membrane can significantly affect the susceptibility of the bacterium to the conjugate by hindering its entry into the bacterial cell through reduced penetration through the phospholipid bilayer. Moreover, as mentioned previously, considering that the primary enzyme target can be either topoisomerase IV or gyrase, the affinity of the PBAE–CIPs for both enzymes can further affect their efficacy in different bacterium types.

To confirm that the observed efficacies were specific to the polymer–drug conjugates and not the polymers themselves, we assessed the activity of the three amine-functionalized polymers (TEGDA-3APD, TEGDA-3AP, and TEGDA-5AP), with no drug attached, to evaluate their potential antimicrobial activity. Less than 50% bacterial growth inhibition was observed at high concentrations of 1 mg mL^–1^ of polymer in both Gram-positive and Gram-negative bacteria (Figure S3).

### Development of Resistance in *P. aeruginosa* and *S. aureus*

**Figure 4:**
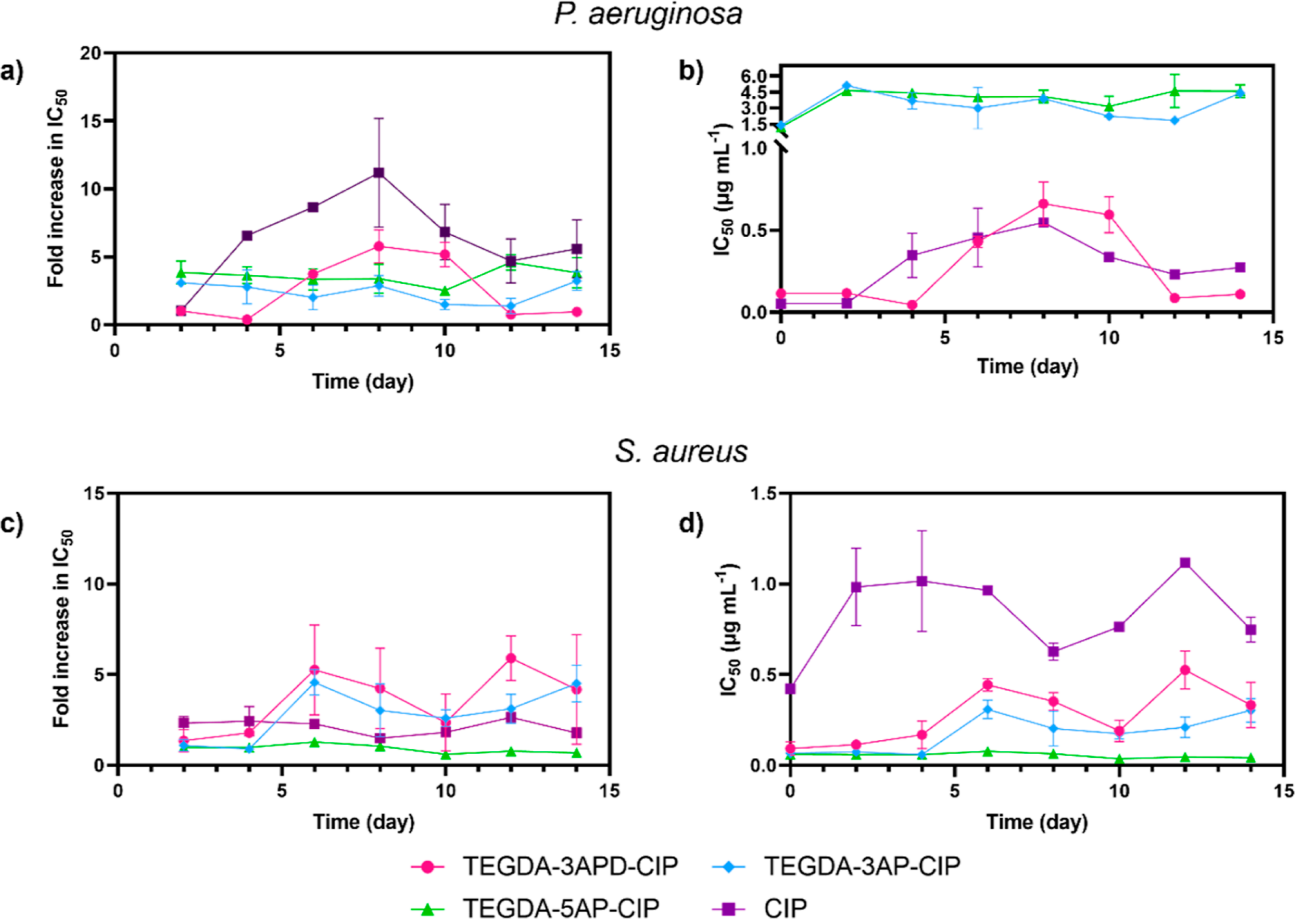
Development of resistance in *P. aeruginosa*and *S. aureus*. (a) Fold increase in IC_50_ values in *P. aeruginosa* of PBAE-CIP conjugates and free CIP following 14 days of continuous exposure; (b) change in IC_50_ (μg mL-1 of CIP attached) values for TEGDA-3APD-CIP (pink), TEGDA-3AP-CIP (blue), TEGDA-5AP-CIP (green), and free CIP (purple) in *P. aeruginosa*; (c) fold increase in IC_50_ values in *S. aureus* of PBAE-CIP conjugates and free CIP following 14 days of continuous exposure; (d) change in IC_50_ (μg mL-1 of CIP attached) values for TEGDA-3APD-CIP (pink), TEGDA-3AP-CIP (blue), TEGDA-5AP-CIP (green), and free CIP (purple) in *S. aureus.* All measurements were performed in duplicate, and the error bars represent the mean ± standard deviation.

In addition to the limited activity of conventional therapeutics in bacterial biofilms, antibiotic efficacy is further hindered by the rapid development of resistance in bacterial cells. We investigated whether attachment to the PBAE scaffold could alter some of the resistance mechanisms present. To analyze this, the fold-change in the IC_50_ against *P. aeruginosa* and *S. aureus* was monitored in cultures cultivated with continuous exposure to the initial IC_50_ concentration of the PBAE-CIPs or free drug for 14 days to maintain evolutionary pressure. As seen from Figure 4a, an increase in the resistance of *P. aeruginosa* to free CIP can be observed following 4 days of continuous exposure, with an average 6.5-fold increase in IC_50_ from around 0.052 to 0.35 μg mL^–1^ reported on day 4. Although resistance was also observed for TEGDA-3APD-CIP over the time frame of the experiment, this was delayed by 2 days compared to that in the free drug, with no resistance to the conjugate observed following 4 days of exposure and the IC_50_ remaining within the 0.11 μg mL^–1^ range. An average 4- and 6-fold increase in the IC_50_ of TEGDA-3APD-CIP was then recorded, following, respectively, 6 and 8 days, to an average IC_50_ of 0.43 and 0.66 μg mL^–1^, demonstrating that resistance to the conjugate does develop in *P. aeruginosa.* Nonetheless, the scale of resistance development to TEGDA-3APD-CIP was comparatively lower than that of free CIP, which showed more than 10-fold increase in the average IC_50_ to 0.55 μg mL^–1^ across 8 days (Figure 4b).

Interestingly, following the initial development of resistance in *P. aeruginosa* against TEGDA-3APD-CIP and free CIP, the bacterium was shown to once again become susceptible to the treatments following 10 days of exposure. This resulted in an average 7-fold decrease in the TEGDA-3APD-CIP IC_50_ between day 10 and day 12, from 0.60 to 0.09 μg mL^–1^, with the effective concentration of the polymer–drug conjugate comparable to that present on day 0. Following 14 days of exposure, *P. aeruginosa* remained susceptible to TEGDA-3APD-CIP with an average IC_50_ of 0.11 μg mL^–1^ observed. For free CIP, following the increase of the average IC_50_ to 0.55 μg mL^–1^ on day 8, the IC_50_ decreased on days 10, 12, and 14 to an average of 0.34, 0.23, and 0.27 μg mL^–1^, respectively. Despite this reduction, the average IC_50_ for free CIP remained 5-fold higher than the initial IC_50_ observed on day 0, resulting in TEGDA-3APD-CIP becoming more effective at eliminating the pathogen than the free drug. The results obtained suggest that the resistance obtained against TEGDA-3APD-CIP and free CIP throughout the initial 8 days of treatment was transient and therefore not maintained in *P. aeruginosa* throughout the duration of the study. Transient resistance to quinolones has been demonstrated previously in literature and suggested to originate from efflux pump overexpression following initial exposure to the treatment. Under normal growth conditions, the expression of genes that encode efflux pumps is generally low. However, in the presence of drugs, there can be a temporary increase in their expression, followed by a decrease, as long as subinhibitory concentrations of the drug are maintained. Thus, we suggest the exposure of the bacteria to TEGDA-3APD-CIP and free CIP caused the activation of efflux pumps resulting in the initial spike of the pathogens’ resistance visible up to day 8. Following 8 days of continuous exposure to treatment concentrations below the IC_50_, the overexpression of efflux pumps was halted to conserve energy.

Considering the limited growth inhibition efficacy of TEGDA-3AP-CIP and TEGDA-5AP-CIP, we did not observe significantly higher resistance development for these two conjugates, likely because of the limited evolutionary pressure exerted on the bacteria by the two polymers.

Comparatively, the increase in IC_50_ observed in *S. aureus* was less significant, with an average 4-fold increase in IC_50_ observed for the TEGDA-3APD-CIP and TEGDA-3AP-CIP polymers and a 2-fold increase in free drug IC_50_ across 14 days of continuous exposure (Figure 4c). The development of resistance was, similarly to *P. aeruginosa*, delayed for the polymer–drug conjugate with an increase in IC_50_ observed on day 6 of exposure, from an average IC_50_ of 0.093 to 0.44 μg mL^–1^ observed for TEGDA-3APD-CIP and an increase from an average IC_50_ of 0.067 to 0.31 μg mL^–1^ for TEGDA-3AP-CIP. For free CIP, an instantaneous spike in IC_50_ was observed between days 0 and 2 from an average IC_50_ of 0.43 to 0.98 μg mL^–1^ (Figure 4d). Interestingly, no resistance to TEGDA-5AP-CIP was observed in *S. aureus* across the 14 day treatment window, with the IC_50_ remaining within the 0.06 μg mL^–1^ range. It is possible that for *S. aureus,* this continued high susceptibility of the pathogen to TEGDA-5AP-CIP resulted from the hydrophobic amine side chain disrupting the bacterial cell membrane to a higher extent than that in the functionalities present in TEGDA-3APD-CIP and TEGDA-3AP-CIP polymers. For all three polymers, even following resistance development to TEGDA-3APD-CIP and TEGDA-3AP-CIP, the IC_50_ values remained significantly lower than the values observed for an equivalent amount of free drug.

Development of resistance to the amine-functionalized polymers with no CIP conjugation was not performed due to their poor efficacy in planktonic bacteria and therefore the limited evolutionary pressure they were expected to exert on the bacterial populations.

Our findings suggest the use of PBAE–CIP conjugates as an appealing alternative to free drug administration as it can delay the development of resistance, therefore extending the length of the therapeutic window. Previous work by Romanovska et al. on CIP conjugation to poly(2-oxazolines) explored the development of resistance to the polymer–ciprofloxacin conjugates, showing a delayed resistance response compared to that of free drug in *E. coli* and *S. aureus*.^25^ Interestingly, this phenomenon was demonstrated to originate from the amphiphilic nature of the polymer chain rather than simply from CIP attachment to the bulky polymer scaffold. Despite initial predictions that the high molecular weight of the polymer attached to CIP would inhibit its transport via efflux pumps, the group demonstrated that the activity of these systems could aid the cell entry of the polymer–drug conjugates, with improved conjugate MICs observed in bacteria with efflux pump overexpression. Further molecular studies are required to understand the mechanisms underlying the resistance development to PBAE-CIPs to assess whether these drug–polymer conjugates can utilize efflux pumps to enter bacterial cells and evaluate their active site binding affinity to both the bacterial gyrase and topoisomerase IV enzymes.

### Polymer–Drug Conjugate Activity against Monospecies *P. aeruginosa* Biofilms

*P. aeruginosa*, currently a global priority pathogen, is linked to some of the most challenging and clinically relevant biofilm infections, including chronic wounds and the cystic fibrosis lung, characterized by reduced production of virulence factors and presence of high-level antibiotic resistance.^59,60^ Hence, the elimination of established *P. aeruginosa* biofilms in vulnerable patients has been frequently unsuccessful even with the use of antibiotics high above their reported minimal inhibitory concentrations.^61^ Moreover, the development of new therapies is hindered by variation between in vitro models with substantial differences in microbial sensitivity and tolerance observed.^62^ Hence, the evaluation of new therapeutics on several in vitro models improves the understanding of their behavior within the biology of the infection, therefore enabling the development of more efficacious treatments.

**Figure 5:**
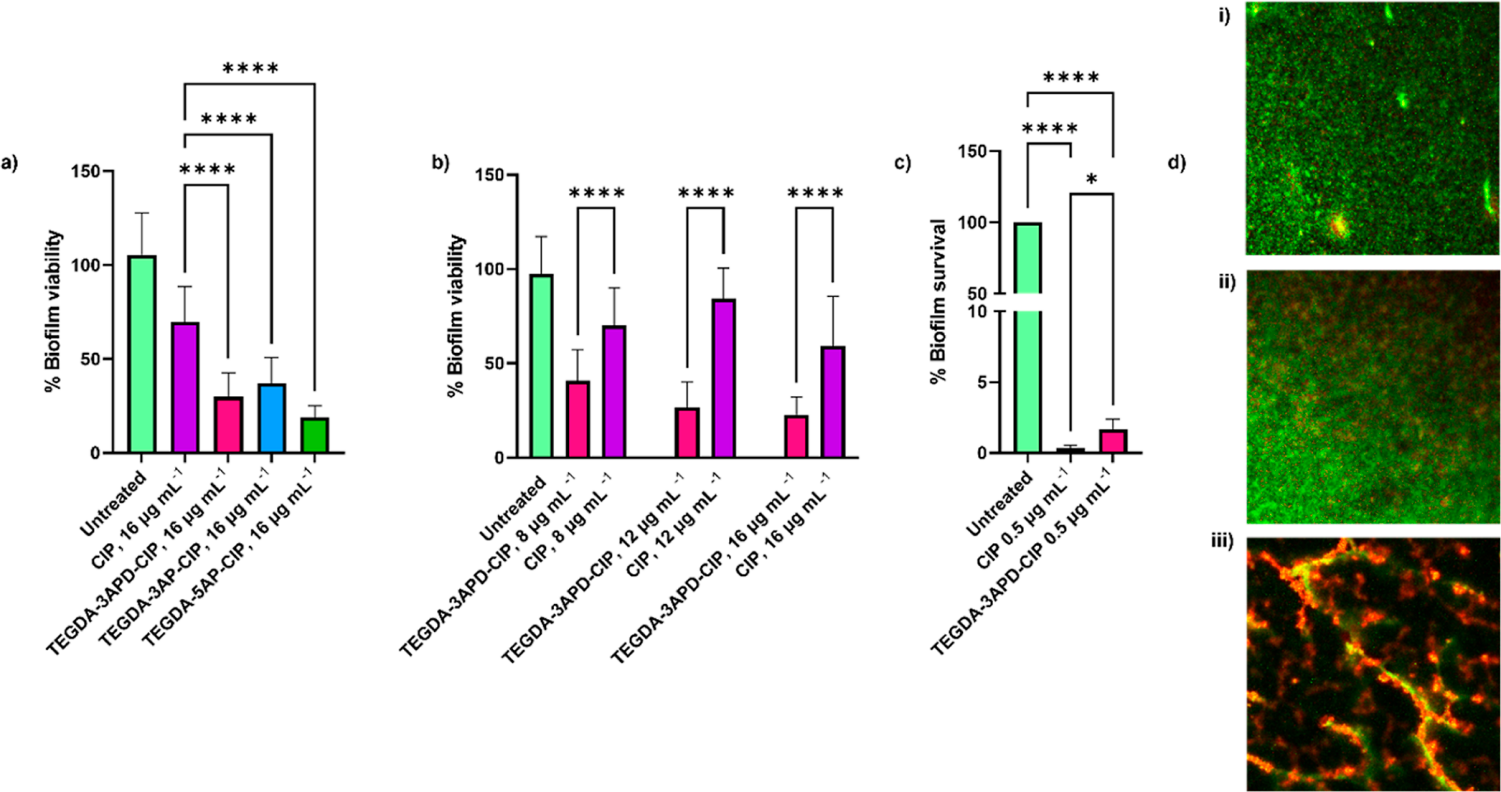
Reduction in viability of rolling bioreactor monospecies *P. aeruginosa*biofilms. (a) Bar charts showing viability in mature *P. aeruginosa* biofilms grown on a rolling bioreactor quantified after treatment with TEGDA-3APD-CIP (pink), TEGDA-5AP-CIP (green), and TEGDA-3AP-CIP (blue), in comparison to the equivalent concentration of free CIP (purple); (b) bar charts showing viability in mature *P. aeruginosa* biofilms grown on a rolling bioreactor quantified after treatment with TEGDA-3APD-CIP (pink) in comparison to the equivalent concentration of free CIP (purple). (c) Bar charts showing percentage survival determined using cfu mL^–1^ values in static monospecies *P. aeruginosa* biofilms quantified after treatment with 0.5 μg mL^–1^ of TEGDA-3APD-CIP (pink), in comparison to the equivalent concentration of free CIP (purple). (d) Representative confocal images of the treated biofilms: (i) untreated control, (ii) biofilm treated with CIP (16 μg mL^–1^), and (iii) biofilm treated with TEGDA-3APD-CIP concentration equivalent to 16 μg mL^–1^ free drug. All measurements were performed in triplicate, using biologically independent replicates, and the error bars represent the mean ± standard deviation. Statistical testing was performed with a one-way ANOVA followed by a posthoc Tukey test to identify individual comparisons. Statistical significance is represented as *p < 0.05, **p < 0.01, ***p < 0.001, ****p < 0.0001.

To evaluate the suitability of TEGDA-3APD-CIP, TEGDA-3AP-CIP, and TEGDA-5AP-CIP polymers as antibiofilm agents, we first tested the PBAE–CIPs on *P. aeruginosa* biofilms grown in dynamic, flow conditions using a rolling bioreactor, resulting in the establishment of thick, mature biofilms. Biofilms were grown for 48 h including a 24 h exposure time to the treatment conditions, followed by the assessment of biofilm viability through live/dead staining. The activity of the three conjugates was measured at concentrations equivalent to 16 μg mL^–1^ of free CIP and compared to that of an unconjugated CIP control. A reduction of over 60% in biofilm viability was observed for the three polymer–drug conjugates, with a 70% reduction for TEGDA-3APD-CIP, 63% for TEGDA-3AP-CIP, and an 81% reduction for TEGDA-5AP-CIP, compared to the free antibiotic control, with an average reduction in biofilm viability of 30% (Figure 5a). We then selected TEGDA-3APD-CIP, the polymer with the highest efficacy in *P. aeruginosa* planktonic cultures and tested its antibiofilm activity against free CIP at three concentrations of 8, 12, and 16 μg mL^–1^ (Figure 5b,d). In each case, the viability of the biofilm was significantly reduced by the conjugate (55% average reduction at 8 μg mL^–1^, 73% reduction at 12 μg mL^–1^, and 77% reduction at 16 μg mL^–1^), compared to the free drug control (30% average reduction at 8 μg mL^–1^, 16% reduction at 12 μg mL^–1^, and 41% reduction at 16 μg mL^–1^), demonstrating that CIP conjugation to a polymer backbone can improve antibiofilm activity. The comparable reduction of biofilm viability observed at the three concentrations of free drug tested was hypothesized to originate from the high resistance of the biofilm to the CIP treatment, thus restricting the efficacy of the antibiotic. We have previously observed that CIP concentrations as high as 60 μg mL^–1^ had limited activity against mature biofilms, grown using a rolling bioreactor, achieving less than a 50% reduction in biofilm viability.^6^ Therefore, the free CIP concentrations applied within this study were not expected to significantly affect biofilm survival.

To evaluate the activity of TEGDA-3APD-CIP in an additional in vitro model, we then measured drug–polymer conjugate activity in a static *P. aeruginosa* biofilm model at a concentration equivalent to 0.5 μg mL^–1^ of free CIP and compared it to a free CIP control at the same concentration. Interestingly, we observed the static biofilms to be significantly more susceptible to much lower PBAE-CIP and free CIP concentrations compared to the biofilms grown using the rolling bioreactor, with less than 10% biofilm survival observed for both the free drug and the polymer–drug conjugate. This was also surprising considering the concentration applied onto the static biofilms was 32 times lower than the one applied on the rolling bioreactor biofilms (Figure 5c). It is likely that this was a result of the thinner and sparser nature of the static biofilm, increasing ciprofloxacin penetration and therefore resulting in biofilm elimination without the assistance of the polymer chain.

For nonfunctionalized TEGDA polymers, a nonsignificant reduction in biofilm viability was observed for the TEGDA-3APD and TEGDA-3AP polymers and an average 20% reduction for TEGDA-5AP (Figure S5). The low antibiofilm efficacy of the nonfunctionalized polymers confirmed that the reduction in biofilm viability originated from CIP conjugation to the polymer chain. The findings correlate with the results observed by Du et al. for PEG–tobramycin, where their conjugate also performed less well than the free drug in planktonic cultures but improved upon free antibiotic efficacy in *P. aeruginosa* biofilms.^22^ In the case of tobramycin, this improvement in antibiofilm activity was attributed to the prevention of tobramycin retention on the biofilm surface through its interactions with the biofilm matrix.^63^ Interestingly, CIP, which is zwitterionic at natural pH and becomes positively charged at lower pH values through amine group protonation,^64^ has been shown to penetrate the *P. aeruginosa* biofilm to a higher extent.^63,65^ In our system, CIP was conjugated to the polymer via a chloroacetyl precursor (CIP-Cl) with acylation of one of the piperidine nitrogens; thus, the predominant positive charges on the polymer–CIP conjugates were from the PBAE backbone. Measurements of the p*K*_a_ of the prepared polymers (Figure S6) confirmed that the PBAE components were the main contributors to the observed basicity, with p*K*_a_ values of ∼6 observed for all polymers treated. These data also demonstrated some buffering capacity of PBAE-CIPs, suggesting they might be protonated in regions of the biofilms exhibiting low local pH and thus might interact with, or penetrate, bacterial membranes in these environments. Experiments in which the pH was measured at different regions in *P. aeruginosa* flow biofilms with nanosensors have indicated heterogeneities in the environments, with regions of lower pH downstream of microbial colonies. However, the measured pH values for the biofilms in this study (albeit at lower spatial resolution) were ∼7.1 for the biofilm interior, suggesting that the polymers would be partially, but not completely, protonated in the regions we tested. It is thus likely that, although the polymers themselves exhibited low antibacterial efficacy, the improved antibiofilm activity of the conjugates was not predominantly via a charge-mediated mechanism. Indeed, resistance to phenotypic adaptations toward persistence within the bacteria was shown to appear only 1 h after biofilm exposure to CIP^66^, which implies that internalization of the drug is a rate-limiting factor. In turn, this supports the hypothesis that polymer conjugation improved delivery into the bacteria but one cannot rule out the role of partial charge on the polymers in helping to achieve this. We therefore suggest that the attachment of the antibiotic to the PBAE might improve the activity within the bacteria of CIP via a different mode of entry or reduced efflux from the bacterial cell walls. These data are also in accord with the results described above for the delay in development of resistance, even though those were obtained with planktonic bacteria. The pH values measured in the planktonic suspensions were similar to those in the biofilms, with near-neutral pH values of 7–7.5, again indicating partial protonation of the amines in the backbones of the PBAE–CIP conjugates and a likely enhanced entry of CIP to target sites as a result of attachment to the polymers.

### Polymer–Drug Conjugate Activity against Static Multispecies Biofilms

While the use of monospecies biofilms provide a good understanding of therapeutics’ behavior within the biology of the infection, it has now been widely established, with the exception of certain infections and laboratory flasks, that the majority of biofilms are composed of multiple species, coexisting with one another.^62^ This is of particular importance when designing treatments for persistent biofilm infections such as those present in the cystic fibrosis (CF) lung. CF biofilms are commonly associated with *P. aeruginosa* and *S. aureus* presence and have been further shown to coexist with fungi such as *Aspergillus* and *C. albicans*.^8^ Hence, to validate our PBAE–CIP polymers as an effective antimicrobial platform, we evaluated our most active polymer (TEGDA-3APD-CIP) into a static multispecies biofilm model.

**Figure 6:**
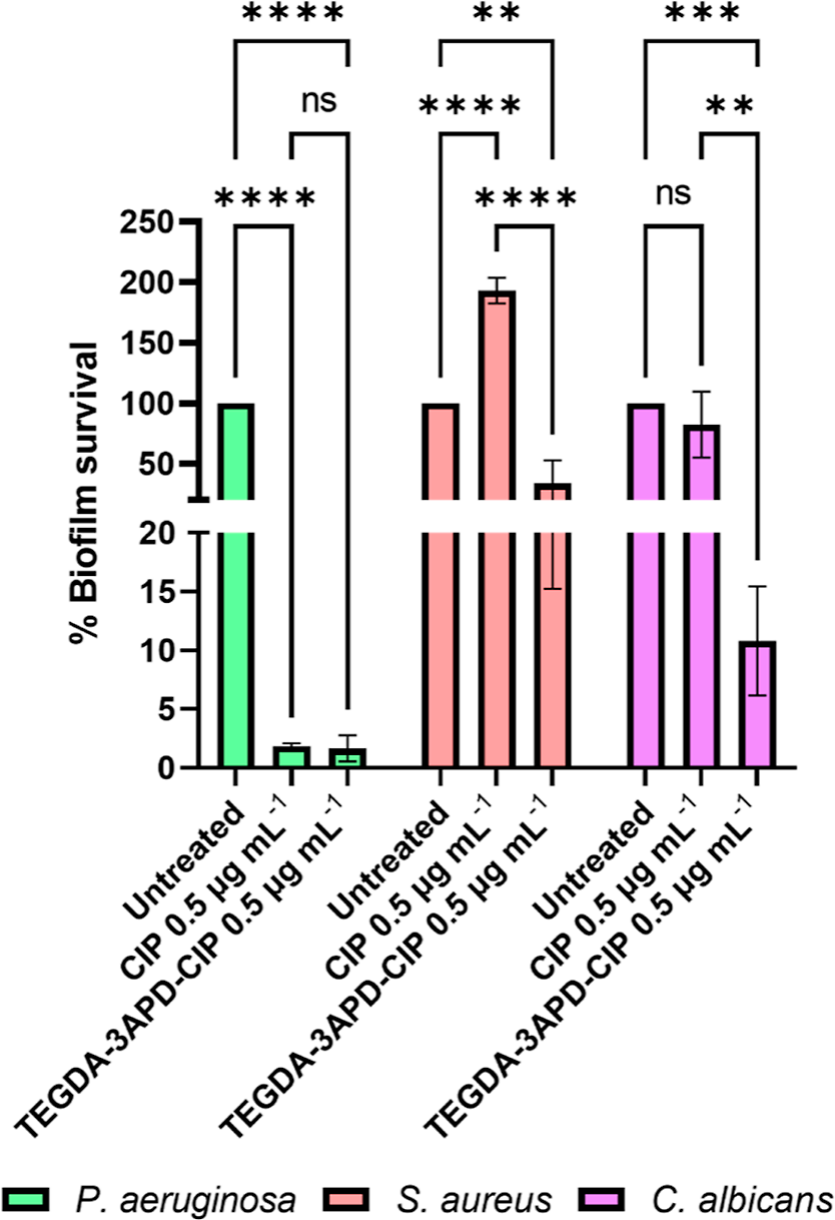
Reduction in static multispecies biofilm viability. Bar charts showing percentage survival determined using cfu mL^–1^ values in static multispecies biofilms composed of *P. aeruginosa* (light green), *S. aureus* (light red), and *C. albicans* (violet) quantified after treatment with TEGDA-3APD-CIP in comparison to the equivalent concentration of free CIP (0.5 μg mL^–1^). All measurements were performed in triplicate, using biologically independent replicates, and the error bars represent the mean ± standard deviation. Statistical testing was performed with a two-way ANOVA followed by a posthoc Tukey test to identify individual comparisons. Statistical significance is represented as *p < 0.05, **p < 0.01, ***p < 0.001, ****p < 0.0001.

Free CIP and TEGDA-3APD-CIP at concentrations equivalent to 0.5 μg mL^–1^ of free drug were tested on a multispecies static biofilm composed of *P. aeruginosa*, *S. aureus,* and *C. albicans*. Interestingly, while the percentage survival of *P. aeruginosa* was comparable between the free drug and TEGDA-3APD-CIP, with less than 10% survival observed in each case, the PBAE–CIP conjugate was shown to be far more efficient at eliminating the other species present (Figure 6). This was particularly visible in *S. aureus*, with the polymer–drug conjugate decreasing survival by over 60%, while free CIP showed a rise in *S. aureus* growth with a nearly 100% increase in the percentage survival. This increase in *S. aureus* growth following treatment with free CIP can be explained by the loss of *P. aeruginosa* providing increased nutrient availability and space for *S. aureus* to dominate the biological niche. Moreover, the elimination of *P. aeruginosa* further resulted in the loss of interspecies quorum sensing molecules such as 2-heptyl-4-hydroxyquinoline *N*-oxide (HQNO) shown to exhibit innate antimicrobial activity against *S. aureus*.^67^ Considering the high efficacy of TEGDA-3APD-CIP against *S. aureus* in planktonic studies, compared to that of free CIP, it is reasonable to assume that the polymer–drug conjugate was more effective at preventing the expansion of *S. aureus* following *P. aeruginosa* elimination.

Free CIP showed low efficacy against the third biofilm microorganism *C. albicans*, with less than a 20% reduction in its survival, while TEGDA-3APD-CIP exposure resulted in a 90% reduction in the fungi’s survival. This surprisingly high efficacy of the polymer–drug conjugate against *C. albicans* may be explained by the killing of *P. aeruginosa* and subsequent release of alkyl quinolones shown to bind to the fungal Topoisomerase II.^68,69^ We suggest that the use of a PBAE–CIP conjugate further enhances the fungal cell wall permeability of CIP, shown to act in synergy with antifungal agents^70^ and therefore promotes the antifungal effect of the released alkyl quinolones. This hypothesis was further verified through the testing of planktonic *C. albicans’* susceptibility to TEGDA-3APD-CIP, free drug, and nonfunctionalized TEGDA-3APD at concentrations equivalent to 0.5 μg mL^–1^ free CIP (67 mg nonfunctionalized polymer) with no efficacy observed for the treatments (Figure S7).

Nonfunctionalized TEGDA-3APD testing demonstrated no activity against the three species analyzed, confirming that the antibiofilm efficacy originated from CIP conjugation to the polymer chain (Figure S8).

### Assessment of Polymer Toxicity in Mammalian Cells

**Figure 7:**
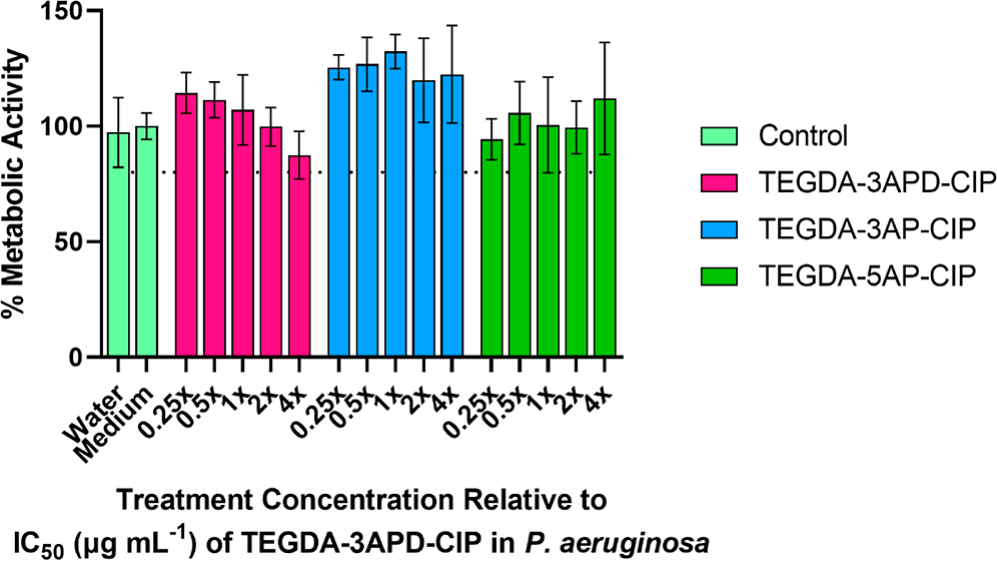
Metabolic activity of A549 cells treated with polymers for 24 h, normalized against killed (0%) and untreated (“medium”, 100%) controls, measured using PrestoBlue assay. Each bar represents the mean and average of two biological replicates each with three technical replicates.

A toxicity assay was performed on A549s, a human alveolar basal epithelial cell line, in order to confirm that the polymers’ toxic effects were specific toward bacterial targets and assess polymer suitability to treat lung-based infections, such as the ones present in the CF lung. TEGDA-3APD-CIP, TEGDA-3AP-CIP, and TEGDA-5AP-CIP were introduced into the medium of growing cells for 24 h, following which the cells’ metabolic activity was measured as an estimate of survival/growth compared to that of untreated controls, with the tested polymer concentrations based on TEGDA-3APD-CIPs IC_50_ in planktonic *P. aeruginosa*. The results show that even at concentrations above the effective bacterial IC_50_, metabolic activity remains above 80% of the untreated control, similar to the buffer (water) control (Figure 7). This indicates that at all tested concentrations, the polymers were not toxic to mammalian cells.

To evaluate further any adverse effects of the polymers on A549 cells, increased PBAE-CIP concentrations, corresponding to up to 10× TEGDA-3APD-CIPs IC_50_ in planktonic *P. aeruginosa*, were introduced into the medium of growing cells for 48 h, after which the metabolic activities of the cells were measured as an estimate of survival/growth and compared with that of untreated controls. Once again, the polymers were shown to be of low acute toxicity to mammalian cells with metabolic activity for all treatments above 80% (Figure S9). This was further verified with bright-field microscopy images taken immediately prior to the PrestoBlue assay (Figure S10) with comparable cell density and shape observed for the PBAE-CIP treated and untreated cells. Additional and more detailed studies, for example, those of membrane disruption and irritancy, would be needed for development toward clinical application, but the initial data indicate that the polymers were well tolerated under the conditions used.

## Conclusions

In conclusion, we have developed a library of PBAE–CIP conjugates with varying central amine content in the PBAE main chain. We then demonstrated an improved efficacy of the PBAE–CIPs in planktonic *S. aureus* compared to that of free drug, while activity against Gram-negative pathogens was limited and lower than that of free CIP. We then demonstrated that our PBAE–CIP conjugates induced a delayed resistance response in both *P. aeruginosa* and *S. aureus*. Considering most bacterial infections are caused by biofilms, we then assessed the activity of the prepared conjugates on mono- and multispecies biofilm models, with improved efficacy demonstrated in each case. Importantly, the PBAE–CIP conjugates performed better in the thicker, more mature biofilms grown in a rolling bioreactor, with substantial differences in the reduction of biofilm viability observed between the PBAE–CIPs and the free drug. In addition, the analogous PBAEs without CIP were either not active against the biofilms or, in the case of TEGDA-5AP, of very low activity, indicating that the mode of action was primarily from the conjugate species rather than via the effects of polycationic species on the polymer chains. The lesser effect of polymer conjugation observed for TEGDA-3APD-CIP in the static monospecies biofilm model was attributed to the reduced thickness and cohesiveness of the biofilm, making it easier for the free drug to penetrate and eliminate the biofilm without the assistance of the polymer chain. Following translation into a multispecies static biofilm model, the polymer conjugate TEGDA-3APD–CIP showed superior killing of all the biofilm components, compared to that of the free drug, once again demonstrating the effectiveness of the PBAE–CIP platform. The differences in free drug and PBAE–CIP activity observed between the two biofilm models used highlight the importance of assessing new antimicrobials in a range of models, with the aim of achieving a better understanding of their behavior within the biology of the infection.

The data demonstrated in the above study show promise in terms of PBAE–drug conjugate applications to treat bacterial infections. Nonetheless, further genomic analysis of the processes taking place within the bacteria following exposure to the conjugates could provide greater detail about the mechanisms behind the activity observed. This is particularly the case for the surprisingly high efficacy of TEGDA-3APD-CIP observed in planktonic *P. aeruginosa*, which may originate from different binding affinities of the three polymer types to CIP target enzymes topoisomerase IV and gyrase. Moreover, further insight into PBAE–CIP use of efflux pumps to penetrate the bacterial cell could shed light on the activity and resistance development observed for the conjugates. Considering the high impact of the substituted amine in the PBAE backbone on conjugate activity, in particular, in Gram-negative bacteria, further exploration of alternative polymer functionalities could be conducted, with the incorporation of more hydrophobic amines and charged molecules being of particular interest. We envisage the use of such developed antibiofilm polymers, following appropriate preclinical studies, being directed toward persistent infections, for example, those in CF lungs, as these are challenging to treat by current methods.

## Data Availability

All relevant data can be obtained upon request from the authors at cameron.alexander@nottingham.ac.uk

## Supporting Information Available

NMR spectra and SEC of polymers, antibacterial efficacy data, pH titrations of PBAE-backbone materials, cell metabolic activity assays, and microscopy images (PDF)
