# Supplementary material for: Ciprofloxacin Poly(β-amino ester) Conjugates Enhance Antibiofilm Activity and Slow the Development of Resistance
Source: ACS Appl Mater Interfaces. 2024 Jan 30;16(5):5412–25. doi: 10.1021/acsami.3c14357 (PMC10859900; doi:10.1021/acsami.3c14357)
Supplement: Supplementary file 1 — am3c14357_si_001.pdf [file am3c14357_si_001.pdf]

Supporting Information

Ciprofloxacin poly(β-amino ester) conjugates enhance antibiofilm activity and slow the development of resistance.

Karolina Kasza[a,b], Brogan Richards[b], Sal Jones[a], Manuel Romero[b,c], Shaun N Robertson[b], Kim R. Hardie[b], Pratik Gurnani*[d], Miguel Cámara[b], and Cameron Alexander*[a]

[a] Division of Molecular Therapeutics and Formulation, School of Pharmacy, University of Nottingham, NG7 2RD, UK

[b] National Biofilms Innovation Centre, School of Life Sciences, Biodiscovery Institute, University Park, University of Nottingham, Nottingham NG7 2RD, UK

[c]Department of Microbiology and Parasitology, Faculty of Biology-CIBUS, Universidade de Santiago de Compostela, Santiago de Compostela, 15782, Spain.

[d]UCL School of Pharmacy, University College London, 29-39 Brunswick Square, London, WC1N 1AX, UK.

E-mail: cameron.alexander@nottingham.ac.uk; P.gurnani@ucl.ac.uk.

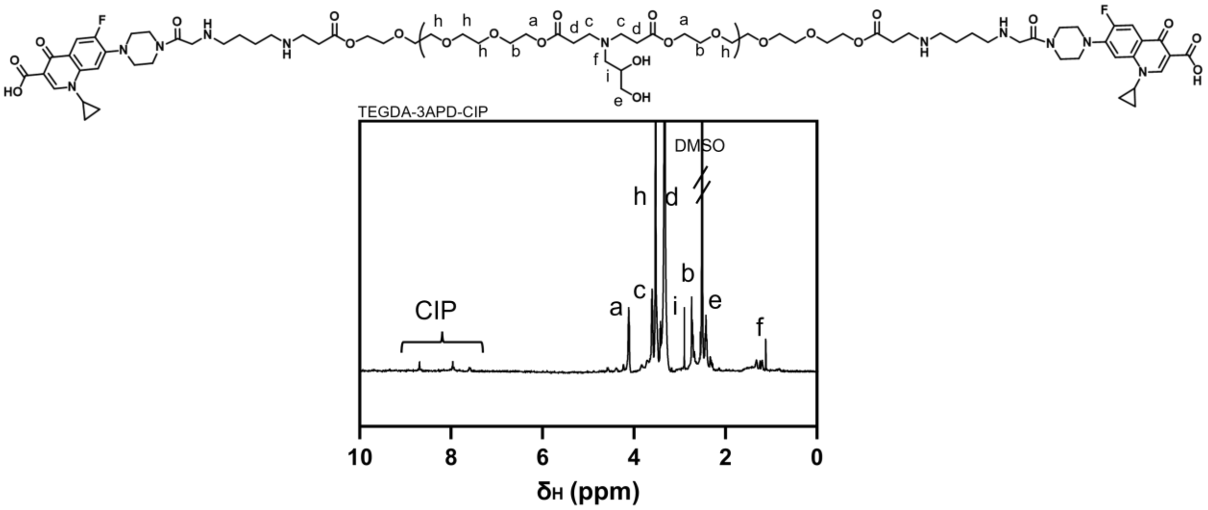
TEGDA-3APD-CIP
DMSO
CIP
h
d
a
c
b
i
e
f
10
8
6
4
2
0
δH (ppm)

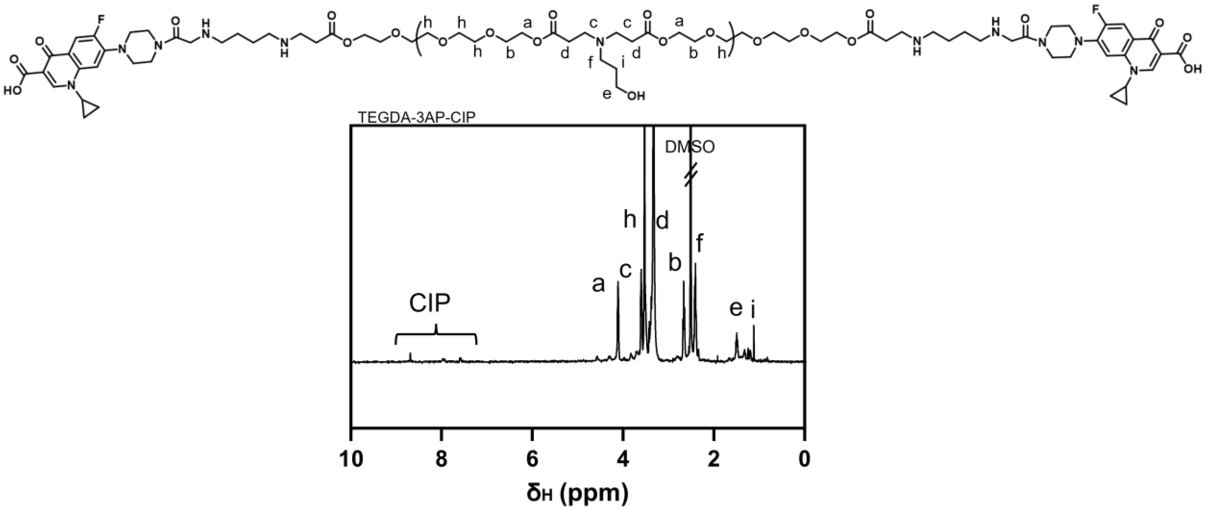
TEGDA-3AP-CIP
DMSO
CIP
h
d
a
c
b
f
e
i
10
8
6
4
2
0
δH (ppm)

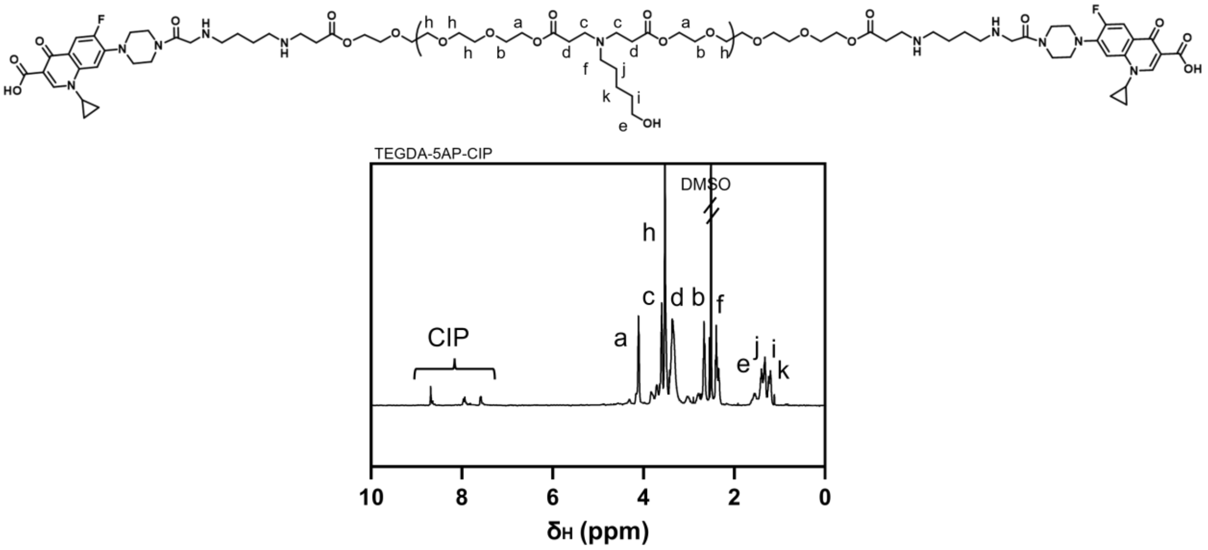
TEGDA-5AP-CIP
DMSO
CIP
h
c
d
b
f
a
e
j
i
k
10
8
6
4
2
0
δH (ppm)

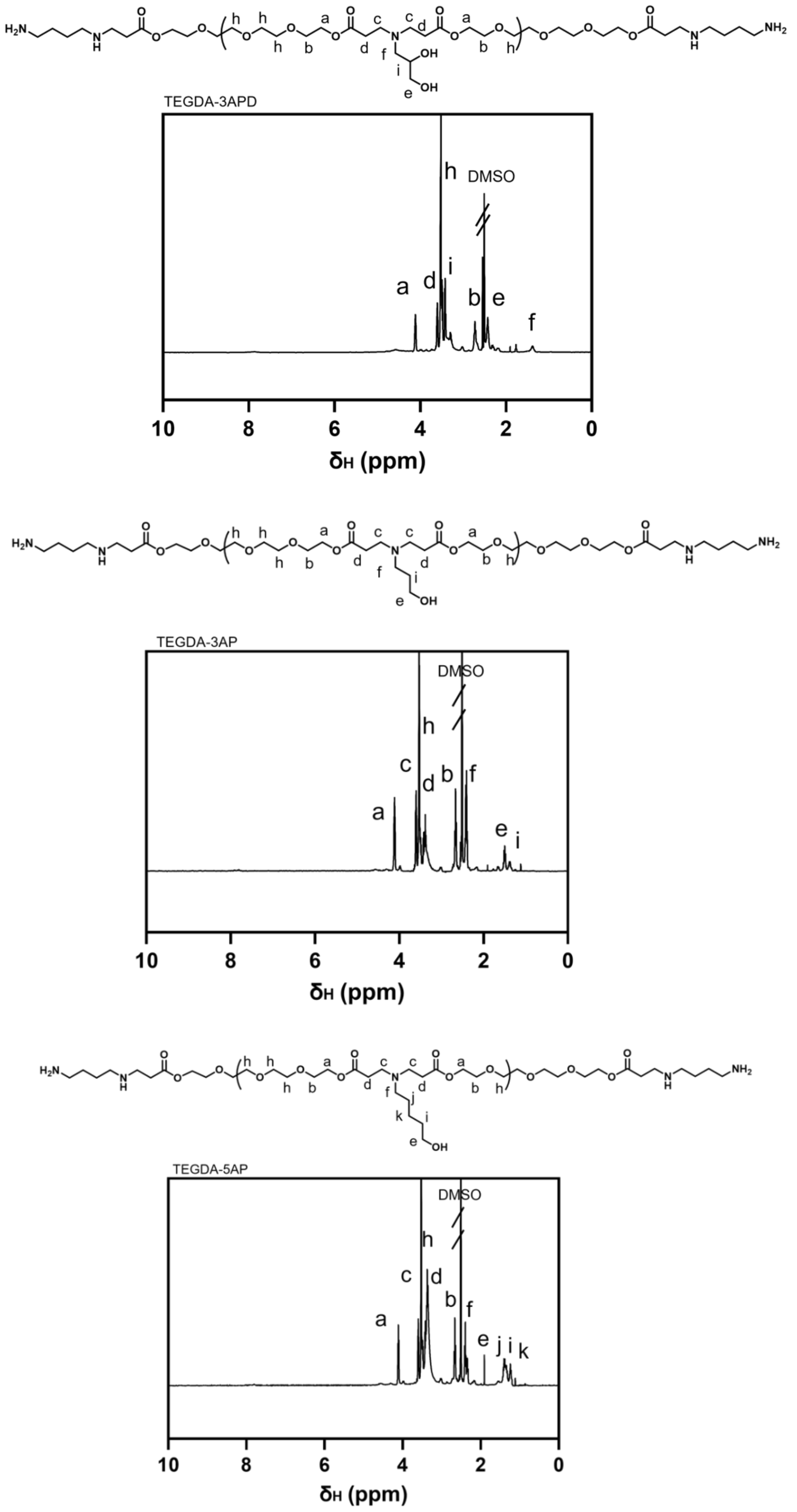

Figure S1: ^{1}H NMR spectra of polymers in DMSO-d6 recorded at 25°C with a Bruker NMR400 MHz.

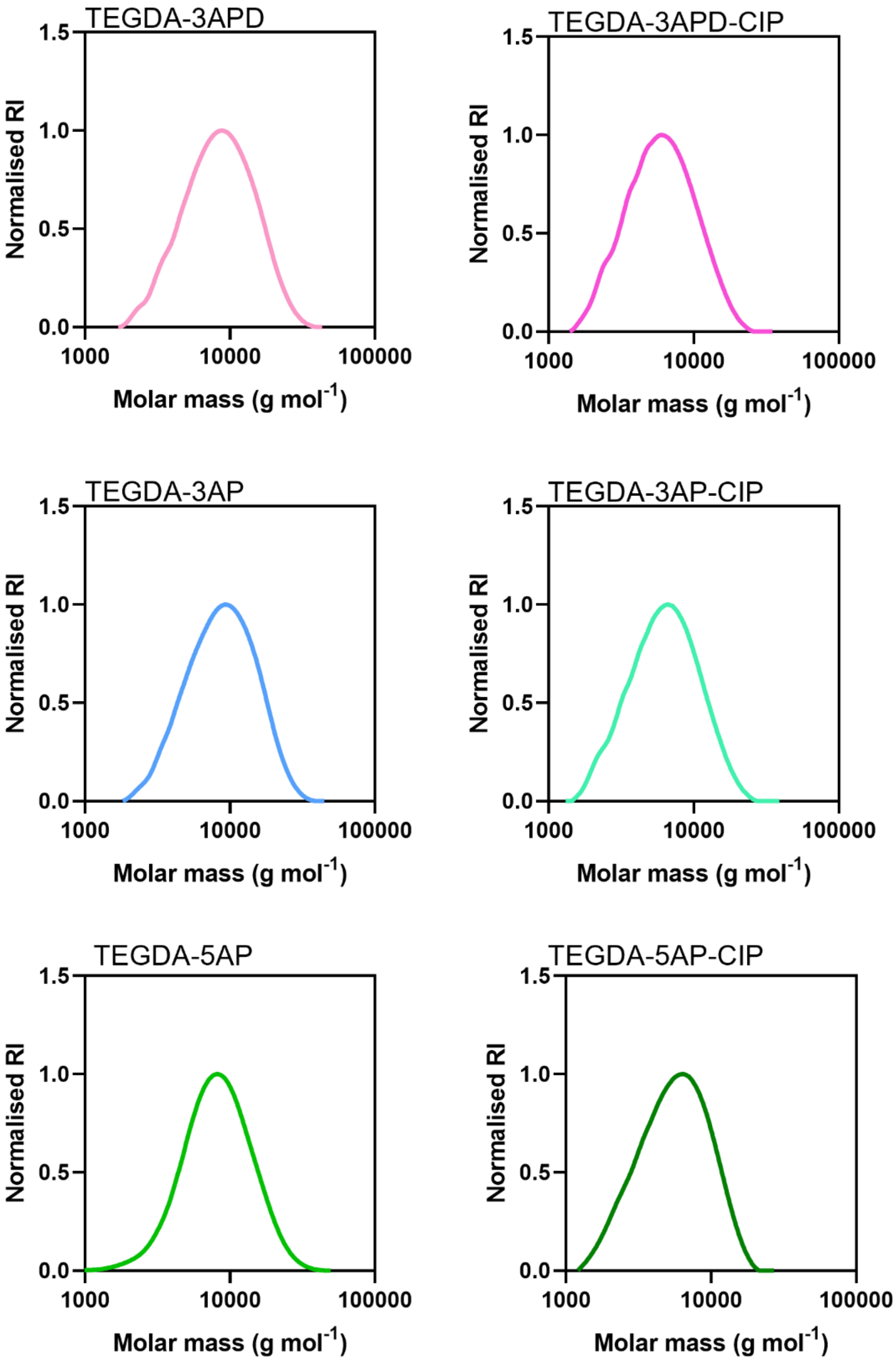

Figure S2: DMF-SEC chromatograms of prepared polymers.

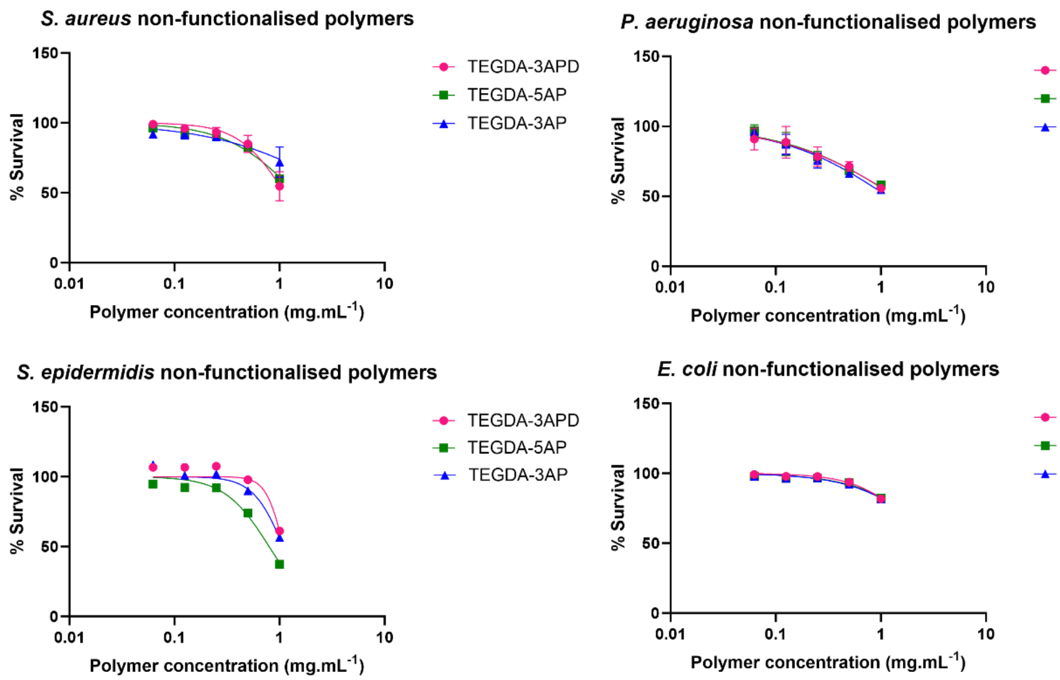

Figure S3: Concentration curves for TEGDA-3APD (pink), TEGDA-5AP (green) and TEGDA-3AP (blue) polymers with no CIP conjugated, against Gram-positive bacteria: *Staphylococcus aureus* (*S. aureus)* and *Staphylococcus epidermidis* (*S. epidermidis)* and Gram-negative bacteria: *Pseudomonas aeruginosa* (*P. aeruginosa*) and *Escherichia coli* (*E. coli*) All measurements were performed in duplicate, using biologically independent replicates and the error bars represent the mean ± standard deviation**.**

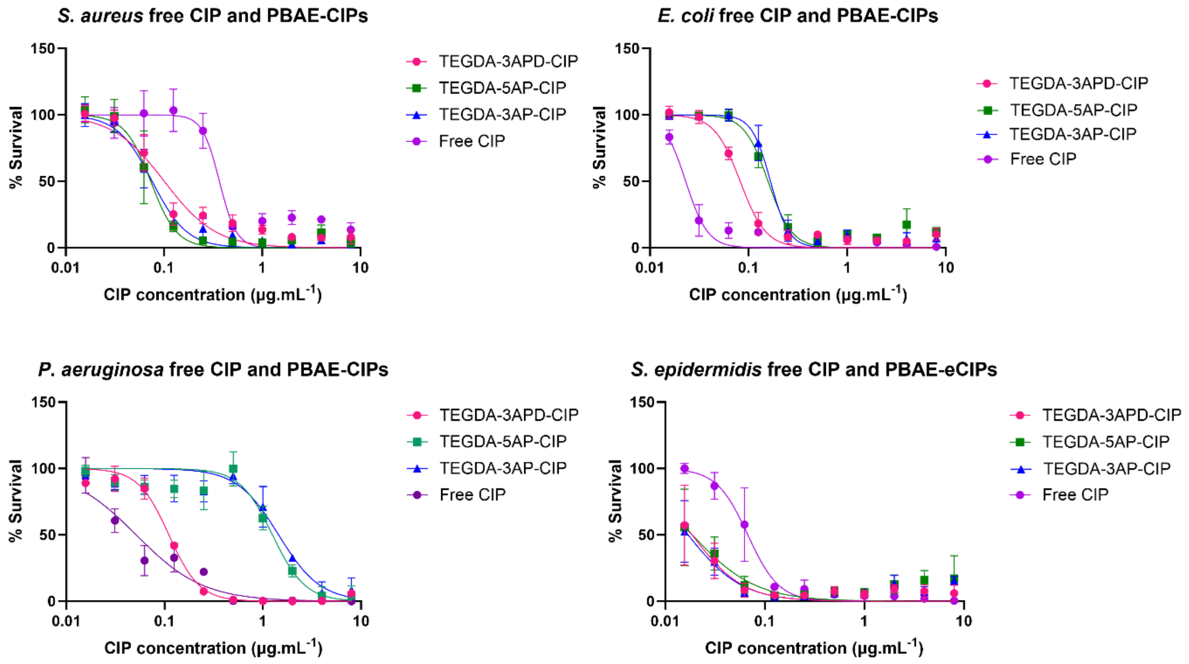

Figure S4: Concentration curves for TEGDA-3APD-CIP (pink), TEGDA-5AP-CIP (green), TEGDA-3AP-CIP (blue) and CIP (purple), against Gram-positive bacteria: *Staphylococcus aureus* (*S. aureus)* and *Staphylococcus epidermidis* (*S. epidermidis)* and Gram-negative bacteria: *Pseudomonas aeruginosa* (*P. aeruginosa*) and *Escherichia coli* (*E. coli*) All

measurements were performed in triplicate, using biologically independent replicates and the error bars represent the mean ± standard deviation.

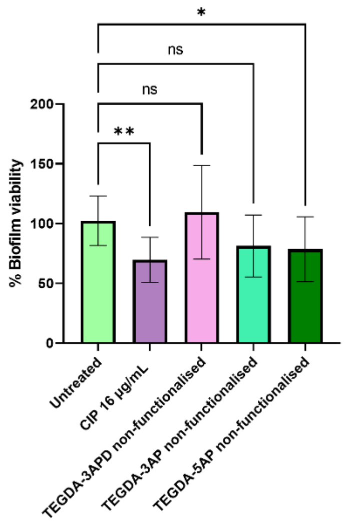

Figure S5: Bar charts showing viability in mature *P. aeruginosa* biofilms grown in a rolling bioreactor, quantified after treatment with TEGDA-3APD (pink), TEGDA-5AP (green) and TEGDA-3AP (blue), at a polymer concentration of 2 mg mL^{-1}. All measurements were performed in triplicate, using biologically independent replicates and the error bars represent the mean ± standard deviation**.** Statistical testing was performed with a one-way ANOVA followed by a post-hoc Tukey test to identify individual comparisons. Statistical significance is represented as *$p < 0.05$, **p <0.01, ***$p < 0.001$, ****$p < 0.0001$.

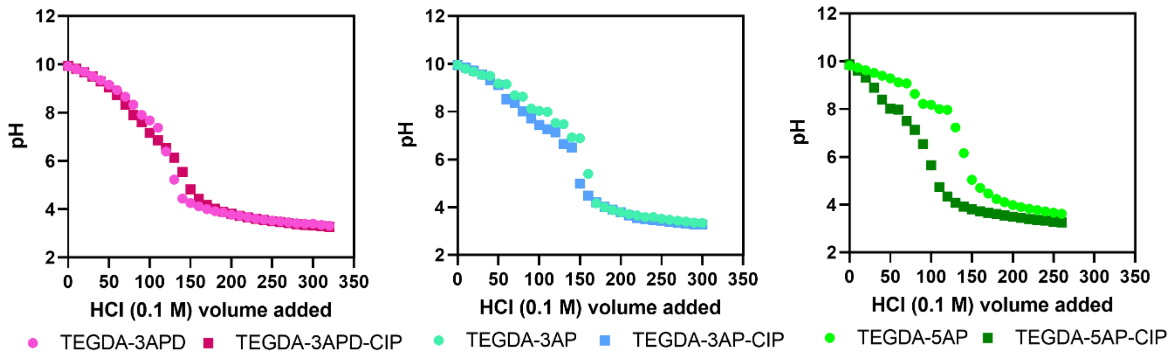

Figure S6: Titration curves obtained by adding HCl (0.1 M) to TEGDA-3APD (light pink circle), TEGDA-3APD-CIP (dark pink square), TEGDA-3AP (light blue circle), TEGDA-3AP-CIP (dark blue square), TEGDA-5AP (light green circle) and TEGDA-5AP-CIP (dark green square) polymers.

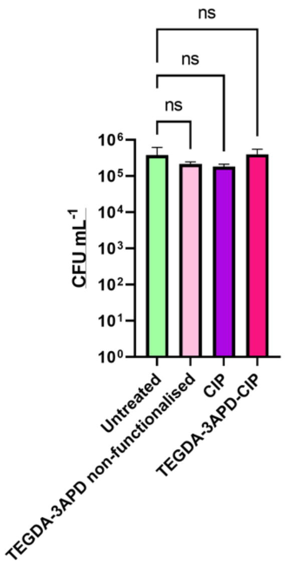

Figure S7: Bar charts showing susceptibility (CFU mL^{-1}) of planktonic *C. albicans* to TEGDA-3APD-CIP (pink), free CIP (purple) and non-functionalised TEGDA-3APD (light pink) at concentrations equivalent to 0.5 µg mL^{-1} free CIP (67 mg non-functionalised polymer). All measurements were performed in triplicate, using biologically independent replicates and the error bars represent the mean ± standard deviation**.** Statistical testing was performed with a one-way ANOVA followed by a post-hoc Tukey test to identify individual comparisons. Statistical significance is represented as **p* < 0.05, ***p* <0.01, ****p* < 0.001, *****p* < 0.0001.

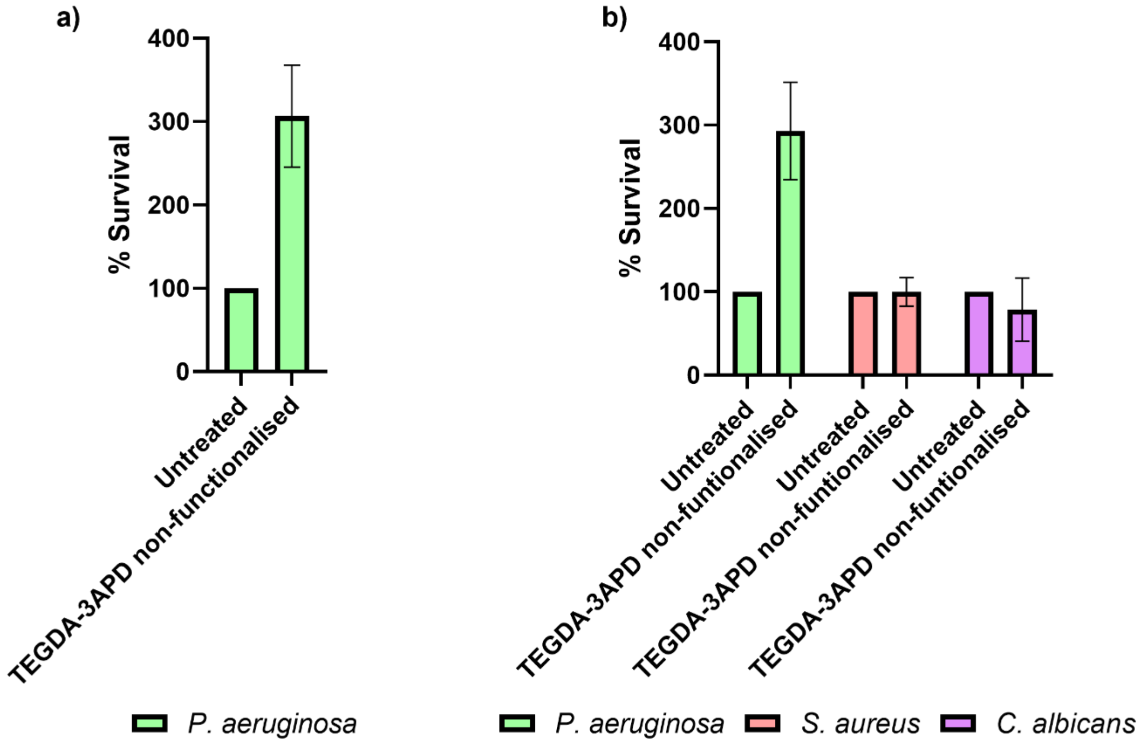

Figure S8: **a)** Bar charts showing percentage survival determined using CFU mL^{-1} values in static monospecies biofilms composed of *P. aeruginosa* (light green) quantified after treatment with 0.068 µgmL^{-1} TEGDA-3APD. **b)** Bar charts showing percentage survival determined

using CFUmL^{-1} values in static multispecies biofilms composed of *P. aeruginosa* (light green), *S. aureus* (light red) and *C. albicans* (violet) quantified after treatment with 0.068 μgmL^{-1} TEGDA-3APD. All measurements were performed in triplicate, using biologically independent replicates and the error bars represent the mean ± standard deviation**.**

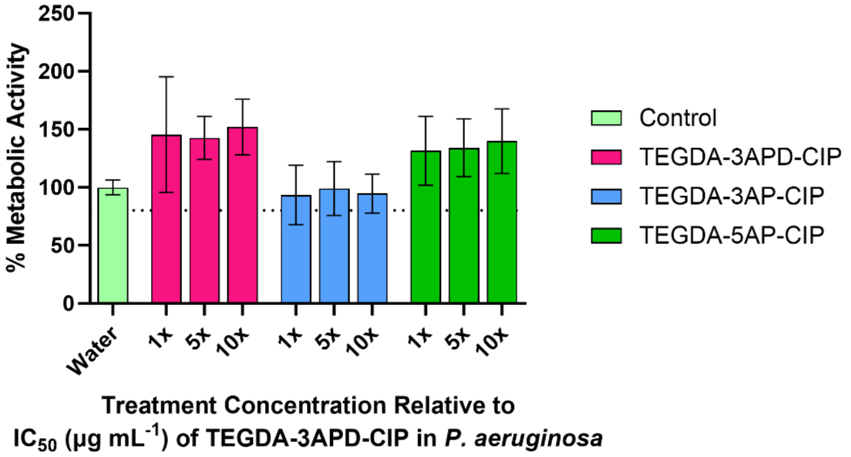

Figure S9: Metabolic activity of A549 cells treated with polymers for 48 h, normalised against killed (0%) and untreated (‘medium’, 100%) controls, measured using PrestoBlue assay. Each bar represents the mean and average of two biological replicates each with three technical replicates.

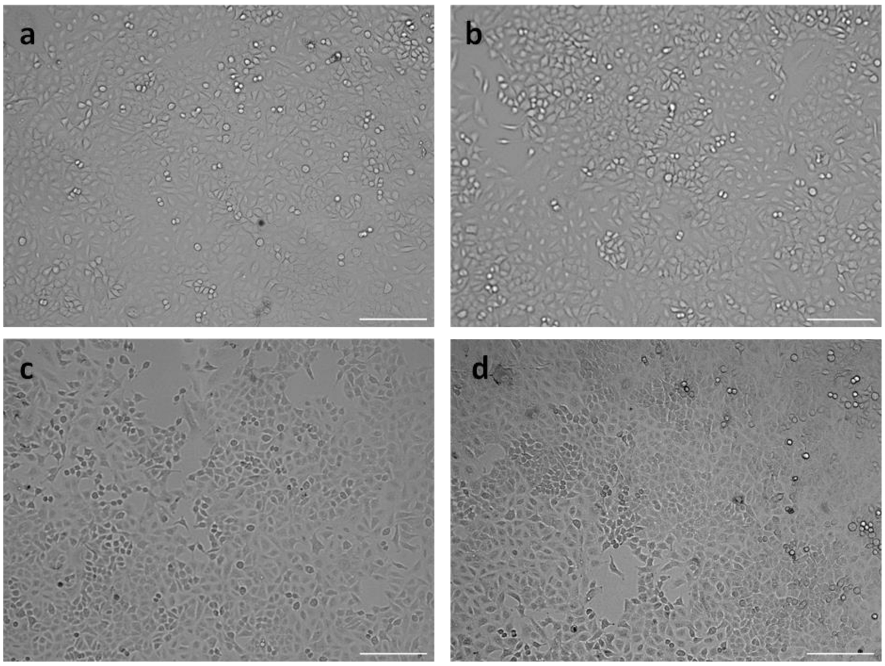

Figure S10: Brightfield microscope images showing minimal density/shape differences between A549 cells following 48h in medium (a) or 10x concentration polymer (b=TEGDA-3APD-CI,P c=TEGDA-3AP-CIP, d=TEGDA-5AP-CIP). Scale bar is 200 μm.
